# *To see or not to see the vet:* A vignette-based study of decision-making by UK dog owners regarding seeking veterinary care for commonly presenting conditions

**DOI:** 10.1371/journal.pone.0339723

**Published:** 2026-01-16

**Authors:** Michelle Farrow, Dan G. O’Neill, Rowena M. A. Packer

**Affiliations:** 1 Clinical Science and Services, The Royal Veterinary College, Hatfield, Herts, United Kingdom; 2 Pathobiology and Population Sciences, The Royal Veterinary College, Hatfield, Herts, United Kingdom; UFSJ: Universidade Federal de Sao Joao del-Rei, BRAZIL

## Abstract

Barriers to accessing veterinary-care for dog-owners are diverse and dynamic, and widely accepted as major canine welfare threats because of potential non-, under- or delayed treatment. Owner knowledge and perceptions are recognised as key influences on decisions to seek veterinary-care but are currently understudied. This study aimed to explore decision-making by UK dog-owners around seeking veterinary-care for common conditions in dogs and identify influences upon these decisions prior to the introduction of Artificial Intelligence technologies. An online vignette-based survey collected responses from UK dog-owners regarding disorder vignettes (*n* = 3) randomly selected from a bank of *n* = 30, that were developed from anonymised VetCompass clinical histories to describe common canine conditions in general practice. Framed as being the dog-owner for each vignette, participants suggested (i) the condition they believed the dog was affected by, (ii) their perceived urgency for seeking veterinary-care and (iii) information-sources used to influence their decisions. Post-vignette questions explored participants’ real-world dog-health information-sourcing behaviours for their own dog(s). Outcomes modelled included (1) accuracy of condition identification, and (2) appropriateness of urgency assessments compared to consensus from a panel of veterinary surgeons. Analyses included *n* = 5316 vignette responses from *n* = 1772 participants. Epilepsy, kennel cough, flea infestation and osteoarthritis were the most accurately identified disorders whereas mast cell tumour, glaucoma and gastrointestinal foreign body were the least accurately identified. Owners using internet-searching for health-information to aid vignette responses scored higher on accuracy than those who did not. Owners perceived conditions to require veterinary-care less urgently than veterinary surgeons in 28.4% of vignette responses; however, using online dog-health groups for information-sourcing reduced the risk of under-urgency. Owners were most accurate at identifying conditions with external clinical signs but often underestimated urgency of seeking veterinary care. Improved accessibility and utilisation of veterinary triage services, telemedicine and information-prescriptions for owners could improve canine welfare by supporting better dog-owner decision-making and optimising veterinary professional workload.

## Introduction

The UK is home to an estimated 10.6 million pet dogs, with approximately 28% of adults owning a dog [1]. There is increasing evidence on the range and frequency of health problems experienced by UK dogs throughout their lifetime, with major disorder risk factors including age, sex and breed [2–4]. The 2006 Animal Welfare Act places a legal responsibility (duty of care) on dog owners to protect their dog from avoidable pain, suffering, injury and disease [5]. Veterinary surgeons are the only group of people that can legally diagnose and treat animals in the UK [6]. Consequently, there are legal, welfare, social and ethical responsibilities on dog owners to appropriately seek veterinary advice and care when health problems arise in their dog, along with veterinary responsibility for reaching an appropriate level of diagnosis and creating a contextualised treatment plan that can be implemented with owner consent [7,8].

Barriers to seeking and accessing veterinary care are diverse, and can be broadly categorised [9] as socioeconomic (e.g., the most frequently reported barrier to accessing veterinary care is financial limitations [10]), geographic (e.g., inequitable geographic distribution of veterinary practices is acknowledged as a potential barrier to veterinary care [11]), and knowledge-based (e.g., the cognitive understanding of owners regarding how to meet their dog’s welfare needs, which includes awareness of when veterinary care should be sought [12]). Despite veterinary practices being an essential source of both practical veterinary care as well as general veterinary advice to owners, a recent survey of UK pet caregivers reported that the internet was the most frequently used information source for pet health, used by 78.6% of UK pet owners [13]. Veterinary professionals have expressed concern about owners using the internet for information on their pet’s health, with online misinformation perceived as a threat to animal welfare [14]. However, the nature, scale and likelihood of these threats from internet searching for healthcare information are unknown. Furthermore, there is poor understanding on what drives owners towards alternative information sources over seeking advice from veterinary professionals.

The current cost of living crisis is a major source of concern for dog owners and may affect their ability to access veterinary care [15,16]. Failure to appropriately access veterinary care is considered a complex societal problem with many causes, with financial constraints considered a major barrier [17]. A recent UK survey reported that around half (48%) of dog owners considered that high and escalating costs of veterinary care were their biggest financial canine concern for the coming year [18]; however, a 2021 US study of dog owners reported that employment and household income were not significantly associated with willingness to seek veterinary care [19]. A veterinary consultation in UK first opinion care is reported to cost in the region of £40 – £60 [20], and the average UK canine pet insurance claim increased from £817 per claim in 2020 to £848 in 2021 [21]. There are several UK charities that may be able to help some owners overcome the financial barriers of veterinary care costs, but financial aid is dependent on owners meeting specific criteria, and many owners who may fail to meet these criteria may still be unable to afford private veterinary care [22–27].

Increasing costs of veterinary care are suggested to be driving owners towards seeking and instituting self-directed treatment at home to manage canine health problems. For example, the Royal Society for the Prevention of Cruelty to Animals (RSPCA) Kindness Index Report 2022 identified dramatic increases in the number of online searches using phrases that suggest that owners are trying to treat their dogs at home without veterinary advice or intervention [28]. The number of searches for the question ‘Can I give my dog paracetamol?’ rose 253% from 15,753 average monthly searches in 2019–39,840 in April 2022. Other common search terms reported included ‘Does my pet need a vaccination’ and ‘Cheap vets near me’ [28]. With the internet offering a low cost, readily available source of information, increasing numbers of owners may defer to the internet over time for canine health information if financial concerns remain, or even grow, as a barrier to accessing appropriate veterinary care by the dog-owning public.

In addition to practical barriers such as financial challenges for accessing veterinary care, there are also perceptual barriers, such as public perceptions and trust in the veterinary profession and changing public value placed on veterinary advice and care [29,30]. In recent years, maintaining the provision of an adequate national veterinary service across the UK has come under unprecedented pressures from trying to balance reduced veterinary workforces and working hours against increased service demands due to a dramatic rise in pet ownership following the COVID-19 pandemic and public expectations of what constitutes good veterinary care [31–33]. External challenges such as the impacts from Brexit and social distancing restrictions during the COVID-19 pandemic have contributed to these challenges [34]. Growing public dissatisfaction with the veterinary profession has resulted in half of all veterinary surgeons working in clinical practice reporting that they experienced online abuse in 2021 [34]. Often termed ‘vet bashing’, this phenomenon generally describes online berating or abuse of veterinary professionals or practices [35]. Public ‘vet bashing’ could be a contributing factor to growing mistrust by owners in the veterinary profession that could further dissuade owners from accessing veterinary care for their pets. Although some research suggests that owners still regard veterinary surgeons as the most popular source of dog health advice [36] and the most trustworthy source of pet health information [13], a recent study identified that many owners would “go home and Google right away” following a consultation with their veterinary surgeon, due to a desire for more detailed pet health information [37]. This implies that many owners in the internet age now see veterinary surgeons as just one, and not the only source, of pet health information and that alternative views are commonly sought in tandem even when veterinary advice is accessed. Emerging developments such as Artificial Intelligence (AI) technologies that have emerged for public use in recent years (e.g., ChatGPT in November 2022, Google Gemini in March 2023), are likely to influence owner information-sourcing behaviours in future more and more. Studies to date have largely focused on veterinary professionals’ use of AI platforms (e.g., [38]) or have theoretically discussed applications for owners without collecting empirical data (e.g., [39]); however, the use and impact of AI platforms have yet to be studied in dog owning populations, and requires further research to understand the opportunities and threats they pose to animal welfare.

Other perceptual barriers to accessing veterinary care include how owners perceive and conceptualise their own dog’s health. The breed-related normalisation phenomenon (“normal for the breed”) describes a cognitive bias whereby owners consider clinical signs of breed-related disorders (particularly those related to conformation) as ‘normal’ or indeed representative of ‘good health’ for that breed, whereas the same clinical features would generally be unacceptable in other dog breeds [40,41]. A common example of this ‘normalisation’ phenomenon centres on owners of brachycephalic (flat-faced) dogs who observe that their dog is exhibiting a range of clinical signs related to brachycephalic obstructive airway syndrome (BOAS) but do not consciously accept that these clinical signs constitute a ‘problem’ or health concern for their dog, instead considering BOAS as an accepted ‘normal’ feature of the breed [40,41]. One consequence of normalisation is that veterinary care may not be sought for normalised disorders even when their dog may be suffering. Aging is another recognised perceptual barrier for seeking veterinary care, as some owners accept physical and behaviour indicators of pain as a normal part of their dog getting older, i.e., reluctance to get out of bed [42]. These and other perceptual phenomena threaten dog welfare via deficits and/or delays in seeking and accessing veterinary care and result in owners not fulfilling their legal duty of care to protect their dog from avoidable pain, suffering, injury and disease [42]. However, to date, there has been little research reporting on how owners perceive the veterinary healthcare needs of their dogs, or which conditions necessitate veterinary intervention.

Learning about owner perceptions across a wide range of common disorders in dogs (not just the specific disorders that currently or previously affected their own animal) with regards to their ability to recognise the conditions involved, and their beliefs regarding the need for veterinary care could be achieved through exploration of hypothetical situations. Using vignettes as a research tool is becoming increasingly popular [43], with these methods widely applied in in social sciences and human healthcare research as well as veterinary science [44–51]. Vignettes can be short scenarios in the form of written words or pictures [52]. Effective vignettes in research should be plausible and realistic, including sufficient information for participants to readily understand the scenario being depicted, and aim to elicit instinctive responses to situations [53]. Vignettes allow interpretation of an individual’s thinking and actions in a paradigm that includes the situational context to be explored and any influential variables to be elucidated [53]. In veterinary medicine, vignettes can evaluate an individual’s response to real life or hypothetical case examples, for example, having been previously used to explore veterinary professionals’ and/or stakeholders’ opinions on difficult scenarios [44,48–50].

A US-based vignette study explored the effects of information-sourcing on 5,000 participants’ (from the general public) ability to ‘diagnose’ and triage of a range of common human healthcare conditions [54]. That interventional study explored whether an obligatory internet search intervention for healthcare information affected participants’ responses regarding their self-diagnosis and urgency of seeking medical attention. The study reported that the internet search did not affect participants’ triage assessments (i.e., appropriateness of how urgently medical attention was needed), but did improve their ‘diagnostic’ accuracy (i.e., accurate identification of the condition affecting the individual) [54]. Vignettes offer a powerful tool to aid exploration of the knowledge gaps regarding owner perceptions of common conditions in dogs in terms of accuracy of recognition of disorders and perception of urgency in seeking veterinary advice. Filling this gap could also contribute to a better overall understanding of the many impacts from owners seeking canine healthcare information outside of the veterinary profession, such as impacts on the welfare of the dogs themselves and even existential questions about the future of the veterinary profession itself.

With this background, this vignette-based study aimed to use hypothetical scenarios to explore decision-making by UK dog owners regarding seeking veterinary care for common conditions, and the potential effects of information sourcing upon these decisions.

## Materials and methods

An interactive, experimental-vignette based online survey (“The DogWeb Survey”) was designed to explore UK dog owners’ decision-making regarding accessing veterinary care. The SurveyMonkey^TM^ platform was used to host the survey, which was open from 22nd February to 11th April 2022. Recruitment was achieved by snowball sampling via a wide range of sources, including via social media (predominantly the Royal Veterinary College’s (RVC) social media accounts including Facebook^TM^, LinkedIn^TM^ and Instagram^TM^ as well as dog-related groups on Facebook), and sharing by key stakeholders (including known influencers in the canine industry, i.e., Canine Arthritis Management (CAM)). This was supplemented by posters incorporating a QR code to participate in the survey which were distributed to veterinary practices local to the main author (MF) (S1 Fig in S1 File). Participants were required to be over 18 years of age, resident in the UK and to currently own one or more dogs. Survey responses were limited to the UK to minimise international variation in dog related information-sourcing [55]. Participants gave informed consent, and responses were anonymised to encourage honesty in participant answers [56]. Ethical approval was obtained from the RVC’s Social Science Research Ethical Review Board (URN: SR2021−0215).

The survey included five broad sections:

(1) Vignettes that each described one of 30 commonly diagnosed conditions in hypothetical dogs based on real life cases from the VetCompass database [57]. Participants assessed each vignette on two domains: what condition they believed the dog was affected by, and their perception of urgency for veterinary care.
(2) Health information-sourcing used for each vignette (e.g., information-sources accessed to inform their responses).
(3) ‘Usual’ health information-sourcing behaviours used by the owner for their own dog (e.g., real-world health information-sources accessed outside of their vignette responses, for information relating to their own current dog’s health).
(4) Dog demographics (e.g., characteristics of the participant’s own dog including breed, sex and age).
(5) Participant demographics (e.g., gender, age, income, education, previous dog ownership experience).

The full survey is in the supplementary files (S2 Text in S1 File).

Questions were designed, piloted and refined iteratively amongst the authors. Pilot surveys were conducted with a sample (*n* = 16) of UK dog owners to check survey logic and ensure ease of understanding and comprehension of questions, with the survey updated following the pilot [58]. These pilot responses were not included in the final dataset.

To improve participant experience, ‘survey logic’ was used to ensure participants only answered the questions relevant to them, by directing each participant to the required next questions depending on their answer [59]. The survey started with the vignettes followed by the demographic questions; this strategy was used to increase participant interest by including content related to the study question (as advertised to them in recruitment materials) early in the survey to capture attention. This strategy has been implemented previously with good response rates and minimal missing data [60].

### Vignettes

Each vignette consisted of a clinical scenario that was approximately 50 words in length and included summary information on the dog’s signalment, relevant history and some contextual information, such as the time of day. The full survey design included 30 vignettes (S3 Table in S1 File) representing 30 different conditions identified as commonly diagnosed in dogs via VetCompass [4], with one example vignette below (gastrointestinal foreign body):

“*Alfie has been vomiting every hour through the night. He ate his evening meal yesterday but vomited it all up. This morning he again ate his breakfast, but then vomited it up shortly afterwards. He seems a little quieter than usual. Yesterday, he went on a long walk, and he was off the lead for most of it. Previously he has been a scavenger. It is 8am on a Sunday*.”

Anonymised VetCompass data records were searched using a range of search terms relevant for each condition to identify real cases with comprehensive, in-depth background and detail recorded in the clinical records [4]. The vignettes were created based on ‘stereotypical’ presentations of each condition extracted from the real clinical histories. The 30 featured conditions were chosen to provide variety in the body systems affected and presentation type (i.e., chronic vs. acute vs. emergency presentations). The 30 vignettes were grouped into ten sets of three, with each participant randomly allocated one set of three to reduce participant fatigue. Each vignette set intentionally included varied condition types, such as one acute, one chronic and one emergency condition as categorised by the validating veterinary surgeons (detailed below).

Following initial creation, each vignette underwent a validation process to develop the final vignettes. Veterinary surgeons were recruited via existing contacts and colleagues of the authors from seven different UK-based general practices for the validation process. The veterinary surgeons were all RCVS registered and experienced in small animal general practice. Fourteen practicing veterinary surgeons, ranging from 3–41 years post-qualification reviewed the 30 vignettes. For each vignette, the veterinary surgeons answered: (i) What condition they believe the dog to be affected by (free-text); (ii) Their recommended timeframe for when the caregiver should seek veterinary care (multiple-choice); (iii) The type of condition (acute, chronic or emergency) (multiple choice); (iv) Any additional comments (free-text).

A majority consensus was sought amongst the veterinary surgeons for the name of the condition in each vignette. For each vignette, a minimum threshold of 71% agreement (10/14) amongst the 14 veterinary surgeons was sought for the name of the condition the dog was affected by [61]. Four vignettes initially did not meet this criterion. Two of these (dry eye [keratoconjunctivitis sicca] and glaucoma) were reviewed and amended following consultation with the RVC ophthalmology service. Refinement of the other two vignettes that did not meet the minimum agreement threshold (diabetes and mast cell tumour) was achieved via consultation of further VetCompass data on these conditions and discussion with co-authors.

The most appropriate timeframe for seeking veterinary care (urgency) for each vignette was derived by majority consensus of the veterinary surgeons, and in the event of an equally divided agreement of urgency, the shorter timeframe (i.e., veterinary care sought more urgently) was chosen. This urgency timeframe was used as the comparison threshold for participant responses.

### Vignette assessment by participants

Participants were instructed to approach the vignettes as if they were the owner for each dog. However, to avoid unnecessary additional workload for real-word veterinary practices, participants were instructed not to contact their veterinary practice regarding the vignettes, even if this would have been their typical health information-sourcing response to the same situation in their own dog(s). Participants were only able to respond to the survey questions once, in a single attempt.

For each vignette, participants reported:

1. What condition they believed was affecting the dog (free-text).
2. Their perception of urgency for seeking veterinary advice and care for that dog. Participants chose one timeframe (multiple-choice).
3. The most influential information-source they used in their decision-making for the vignette. Participants chose one information source (multiple-choice).
4. All the information-sources they used while considering their answers for the vignette. Participants selected all information-sources that applied (multiple-choice).

### Post-vignette questions: Usual information-sourcing behaviours

Participants were asked to identify their three most frequently used information-sources in the real-world when making decisions around seeking veterinary care for their own current dog. The list of options was generated from existing literature [13,36]. An ‘Other, please specify’ category was included to account for novel information-sources [32]. Participants were next asked to indicate from multiple choice options the information-source that was the most influential for their decision-making around their own dog’s health. Finally, participants reported the most important factors influencing where and how they usually sought information relating to their dog’s own health from a multiple-choice list, including reputation and affordability.

### Post-vignette questions: Dog and owner demographics and pet healthcare behaviours

Participants provided demographic information on their current dog (or if more than one was owned, their dog whose name started earliest alphabetically) and current healthcare behaviours regarding them. The information collected included breed (which was categorised during data coding to allow evaluation of brachycephalic breed ownership as a risk factor for study outcomes); registration at a veterinary practice; routine veterinary practice visits; non-routine veterinary practice visits; insurance status; vaccination status; flea and worm prevention and long-term conditions affecting their dog.

Owner demographic information collected included: age; sex; location; highest level of education; household income and whether the participant was employed in the canine professional industry (if yes, in what capacity (free-text)). Information on the participants’ dog ownership experiences were also collected, including current dog ownership (e.g., whether they lived in multi-dog households) and their previous dog ownership (e.g., how many years they have personally owned dogs for and how many dogs previously owned).

### Data preparation

Raw survey data were exported from SurveyMonkey^TM^ into Microsoft Excel for manual data cleaning. Responses were excluded from individuals who did not meet the inclusion criteria or that did not include data beyond the consent and inclusion criteria. Where free-text responses were deemed to fit within the scope of existing fixed-choice responses, data were back-allocated into appropriate categories. Where free-text responses were not appropriate for back-coding, an inductive approach was taken to group and further categorise free-text using content analysis techniques [62].

#### ‘Accuracy’ of condition naming.

Free-text responses were mapped to create a list of conditions that would allow quantitative statistical analysis. Each answer was assessed for its ‘accuracy’ and ‘certainty’:

(a) ‘Accuracy’ assessed whether the answer concurred with the original diagnosis from the VetCompass history and that was later validated by the validating veterinary surgeons. Accuracy scored from zero to three using a framework developed specifically for each individual vignette. An accuracy score of zero constituted an answer that was incorrect, a score of one for a partially correct answer (ambiguous terms but generally relevant). A score of two was given for a partially correct answer (using lay terms), and a score of three for a correct response using precise, technical language in line with the original diagnosis.
(b) The responses were assessed for ‘certainty’ using a scale of one to three. A certainty score of one was given for responses that provided no answer or alluded to high uncertainty, e.g., ‘I do not know’ or ‘not sure’, a score of two for answers that indicated some uncertainty via including multiple suggestions or an answer followed by a question mark, or prefixes such as ‘possible’ or ‘maybe’, and a score of three for answers that stated one condition only, with no further indication of uncertainty, regardless of the true accuracy.

The scores for ‘accuracy’ and ‘certainty’ were combined using a multiplicative scoring method (example, S3 Table in S1 File) in line with previous methods in canine welfare research [63] to generate an overall score on a continuous scale from zero to nine. A score of nine represented an accurate, and highly certain answer.

#### ‘Urgency’ of conditions.

Participants’ perceptions of the urgency of veterinary care for each vignette were compared to the veterinary surgeons’ recommended timeframes. Each response was coded as:

(a) More urgent than recommended by veterinary surgeons (i.e., the participant would seek veterinary care sooner than recommended);
(b) Equal to the veterinary surgeons’ recommended timeframe (i.e., the participant would seek veterinary care in the same timeframe as recommended); or
(c) Less urgent than veterinary surgeons recommended (i.e., the participant would seek veterinary care later than recommended).

For analyses, the categories were further collapsed into a binary variable: equal to or more urgent than veterinary surgeons’ recommendation versus less urgent than veterinary surgeons’ recommendation.

### Data analysis

Following cleaning in Excel, data were imported into IBM SPSS Statistics v27 (SPSS Inc, Chicago, IL, USA). Initial data analysis included calculation of descriptive statistics (frequency and percentage). Continuous variables were assessed for normality via visual inspection of histograms, and statistical significance was set at *p* < 0.05.

#### Accuracy analyses.

Median accuracy scores were calculated for each of the 30 conditions. Univariable association between the median accuracy score and the presentation type (i.e., emergency, acute or chronic) was assessed using a Kruskal-Wallis test for non-normally distributed data. Univariable association between the median accuracy score and the primary veterinary care prevalence of disorders was assessed for each condition using Spearman’s rank for non-normally distributed continuous data with prevalence data derived from a study of dogs under UK primary veterinary care in the VetCompass system [4].

Multivariable generalised linear mixed modelling was used to evaluate risk factors for accuracy. Predictor variables (including owner demographics, dog demographics and habitual information-sourcing habits) with univariable association (*p* < 0.01) were taken forward into multivariable analyses. Repeated measures were accounted for by inclusion of vignette block (from 1–10) as a fixed effect. Variables were assessed for collinearity. Model development used manual backwards stepwise elimination. An ‘information theory’ approach was taken that meant variables of a priori key interest were retained in models regardless of their significance level [64], specifically: owner use of the internet as a health information source (both for vignette-specific information and general day-to-day dog health information-sourcing); whether participants’ own dogs were affected by any long-term health conditions, whether participants were first-time dog owners, and whether participants currently owned a brachycephalic purebred/brachycephalic crossbreed dog.

#### Urgency analyses.

The percentage of owners that considered each condition as less urgent than the validating veterinary surgeons was calculated for all 30 conditions (percentage less urgent (%)). Univariable association between urgency as a binary variable and the prevalence of conditions in the vignettes was assessed using Spearman’s rank for non-normally distributed continuous data. Univariable association between urgency and presentation type (i.e., emergency, acute or chronic) were compared using a Kruskal-Wallis (*n* > 2 categories). The same univariable associations were explored as in the accuracy models.

Multivariable binary logistic regression modelling was used to evaluate risk factors for urgency as a binary outcome (less urgent vs. as urgent/more urgent than a veterinary surgeons’ assessment). Predictor variables (including owner demographics, dog demographics and habitual information-sourcing habits) with univariable association (*p* < 0.01) were taken forward into multivariable analyses. Repeated measures were accounted for by inclusion of vignette block (from 1–10) as a fixed effect. Variables were assessed for collinearity. As with the accuracy model, the urgency model development used manual backwards stepwise elimination combined with an ‘information theory’ approach, including the same variables described for the accuracy model.

#### Comparison of accuracy and urgency.

For each condition, associations between median accuracy score and the percentage of participants’ assessments that were ‘less urgent’ was assessed at univariable level using Spearman’s rank testing.

## Results

The study included 2869 participants, with 1097 (38.2%) excluded from the final analysis. Of these, 618 (21.5%) provided no information beyond vignette selection, 412 (14.4%) gave incomplete responses and 67 (2.3%) gave no information beyond initial consent. The final analysis included 1772 participants and 5316 vignette responses (*n* = 3 vignettes per participant) (response per vignette reported in S4 Table in S1 File).

### Owner demographics

The majority of the 1772 participants in the final analysis were female (*n* = 1657, 94.3%). The participants were of an older demographic, with the most common age group being 55–64 years (*n* = 482, 27.5%). The most common household income was £20,001 – £30,000 (*n* = 228, 13%) and the most common highest level of education was level 6 qualification (i.e., degree with honours) (*n* = 493, 28.1%). All UK counties were represented in the study; the county with the most responses was Wiltshire (*n* = 102, 5.8%)

Participants were mostly not first-time dog owners (*n* = 1563, 88.6%), with many participants reporting they had over 16 years’ experience of dog ownership (*n* = 1147, 65%). Most participants did not work within a canine industry (*n* = 1396, 79.3%), but of those who did, the most common job role was dog trainer (*n* = 77, 28.6%) followed by veterinary nurse (*n* = 50, 18.6%). (Table 1).

**Table 1:** Participant demographic information from (*n =* 1772) UK dog owners surveyed.

Variable	Category	Number (n)	Percentage (%)
**Participant gender**	Male	1657	94.3
	Female	80	4.6
	Other	3	0.2
	Prefer not say	18	1.0
**Participant age**	55–64 years old	483	27.5
	45–54 years old	403	23.0
	65–74 years old	281	16.0
	35–44 years old	268	15.3
	25–34 years old	208	11.9
	18–24 years old	51	2.9
	75–84 years old	50	2.8
	Prefer not to say	11	0.6
**Participant annual household income**	Prefer not to say	402	22.9
	£20,001–£30,000	228	13.0
	£30,001–£40,000	178	10.1
	£40,001–£50,000	167	9.5
	£50,001–£60,000	152	8.7
	£10,001–£20,000	142	8.1
	More than £100,001	120	6.8
	£60,001–£70,000	108	6.2
	£70,001–£80,000	78	4.4
	£90,001–£100,000	69	3.9
	£80,001–£90,000	61	3.5
	Up to £10,000	49	2.8
**Highest level of education**	Level 6	494	28.1
	Level 7	404	23.0
	Level 3	328	18.7
	Level 1/2	321	18.3
	Prefer not to say	93	5.3
	Miscellaneous	54	3.1
	Level 8	46	2.6
	Level 5	11	0.6
	Level 4	6	0.3
**1**^**st**^ **time dog owner**	No	1563	88.6
	Yes	202	11.4
**Duration of experience of dog ownership**	Over 16 years	1147	65.0
	12–15 years	159	9.0
	8–11 years	145	8.2
	4–7 years	141	8.0
	1–3 years	136	7.7
	Less than 1 year	36	2.0
	Over 16 years	1147	65.0
**Employment in canine industry**	No	1396	79.3
	Yes	365	20.7
**Canine industry workers’ jobs**	Dog trainer	77	4.6
	Veterinary nurse	50	3.0
	Dog groomer	28	1.7
	Dog behaviourist	27	1.6
	Dog walker	27	1.6
	Animal care assistant	16	1.0
	Dog day-care/boarding kennels	14	0.8
	Veterinary surgeons	13	0.8
	Rehoming centre staff	8	0.5
	Veterinary scientist	5	0.3
	Pet shop worker	2	0.1
	Veterinary physiotherapist	1	0.1
	Veterinary receptionist	1	0.1
**Participants’ UK geographic region (top 10)**	Wiltshire	102	5.8
	Hampshire	66	3.8
	Kent	66	3.8
	Essex	52	3.0
	Somerset	51	2.9
	Lincolnshire	50	2.8
	Lancashire	46	2.6
	Cambridgeshire	44	2.5
	Devon	44	2.5
	Nottinghamshire	43	2.4

### Canine demography lifestyle and veterinary care

Labrador Retrievers was the most owned breed by participants (*n* = 176, 10%), followed by Crossbreeds (*n* = 165, 9.3%) and Cocker Spaniels (*n* = 122, 6.9%). Overall, 7.6% (*n* = 122) of dogs owned by participants were considered a brachycephalic purebred or a brachycephalic crossbreed (Table 2).

**Table 2:** Dog demographics from a survey of (*n =* 1772) UK dog owners.

Variable	Category	*n*	%
**Multi-dog household**	No	636	36.0
	Yes	1123	64.0
**Breed (top 10)**	Labrador Retriever	176	10.0
	Crossbreed	165	9.3
	Cocker Spaniel	122	6.9
	Border Collie	107	6.1
	Golden Retriever	93	5.3
	Beagle	68	3.9
	English Springer Spaniel	57	3.2
	German Shepherd Dog	42	2.4
	Cockapoo	34	1.9
	Staffordshire Bull Terrier	30	1.7
**Dog age**	2	300	17.0
	Less than 1	175	9.9
	5	151	8.6
	4	147	8.3
	3	143	8.1
	7	128	7.3
	8	127	7.2
	6	121	6.9
	10	114	6.5
	9	101	5.7
	11	82	4.6
	12	74	4.2
	13	45	2.5
	14	33	1.9
	15	17	1.0
	16	6	0.3
	18	1	0.1
**Veterinary practice registration**	Yes	1755	99.3
	No – I have not registered them as yet, but I intend to in the future	7	0.4
	No – I have not registered them and do not intend to unless they become ill	4	0.2
	No – I have tried to register them but have been unable to find a practice that will register us	1	0.1
**Routine veterinary practice visits**	Once a year	703	39.9
	Every 6 months	380	21.5
	Less than once a year	253	14.3
	Every 3 months	194	11.0
	Monthly	103	5.8
	Only when necessary	86	4.9
	I don’t routinely take my dog to the vets	27	1.5
	Miscellaneous	12	0.7
	Weekly	3	0.2
	Every 6 weeks	2	0.1
	Once every 2 weeks	1	0.1
**Non-routine veterinary practice visits**	Less than once a year	654	37.1
	Once a year	279	15.8
	Every 6 months	265	15.0
	Only when necessary	184	10.4
	Every 3 months	150	8.5
	Monthly	121	6.9
	Miscellaneous	49	2.8
	I don’t take my dog to the or haven’t needed to yet	43	2.4
	Weekly	8	0.5
	Every 2 weeks	5	0.3
	Every 6 weeks	4	0.2
	Only when necessary	1	0.1
**Insurance**	Yes	1044	59.2
	No – and I do not plan to insure them	359	20.3
	No – they were insured but I have since cancelled or did not renew their policy	154	8.7
	Yes – at the moment, but we are undecided whether to continue with it at the next renewal	105	5.9
	No – they came with 4 weeks free insurance as a puppy, but I did not continue with this	62	3.5
	No – but I plan to insure them in the future	15	0.8
	No – I have never heard of pet insurance	15	0.8
	No – insurers wouldn’t cover the dog	9	0.5
	I am undecided	1	0.1
	No – I wish I had taken out insurance	1	0.1
**Vaccination**	Yes – every year	1086	61.6
	Yes – not every year	561	31.8
	No	116	6.6
**Regular flea and worm prevention**	Yes – I acquire these through my vet	888	50.3
	No – I have chosen not to use anything regularly to prevent fleas and/or worms	282	16.0
	Yes – I acquire these from a shop, but without prescription	243	13.8
	Yes – I use natural/homeopathic remedies	195	11.0
	I only worm regularly	57	3.2
	I prefer to test for flea/worms before treating	49	2.8
	Yes – I acquire a prescription through my vet and purchase items online	25	1.4
	Yes – I purchase items online	22	1.2
	Miscellaneous	2	0.1
	No – but use natural remedies when I do	2	0.1
	I only flea and tick regularly	1	0.1
**Diagnosed with a long term condition**	No	1265	71.7
	Yes	499	28.3

N.B. Owners of multiple dogs answered based upon one dog.

Most of the dogs owned by participants lived within multi-dog households (*n* = 1129, 64%). Almost all dogs were registered at a veterinary practice (*n* = 1755, 99.3%) and an annual visit (*n* = 703, 39.9%) was the most common interval for how often owners presented their dog to a veterinary practice for routine veterinary care (including vaccinations, flea and worming appointments) with half of dogs (*n* = 888, 50.3%) receiving regular prescription flea and worm prevention purchased through their veterinary practice.

For non-routine treatments, the most reported frequency for visiting a veterinary practice was under once per year (*n* = 654, 37.1%). Over half of dogs owned by participants were insured (*n* = 1044, 59.2%) and vaccinated annually (*n* = 1086, 61.6%). Over a quarter of dogs owned by participants (*n* = 499, 28.3%) had been diagnosed with at least one long-term health condition.

### Usual canine health information-sourcing behaviours

When searching for information regarding their own dog’s health, the most influential sources of information stated by owners to aid their decision-making were the owners’ own knowledge (*n* = 754, 42.7%), their local veterinary practice (*n* = 497, 28.1%) and internet searches (*n* = 142, 8.0%). The most frequently used sources of information on canine health information sought out by owners were their own knowledge (*n* = 1300, 73.7%), their local veterinary practice (*n* = 1078, 61.1%) and internet searches (*n* = 869, 49.2%) (Table 3).

**Table 3:** Information-sources used habitually by (*n =* 1772) UK dog owners when deciding whether to seek veterinary care for their dog.

Variable	Category	*n*	%
**The most influential canine health information-sources**	My own existing knowledge/experience	754	42.7
	Directly contact my local vets/veterinary professionals in practice	497	28.1
	Internet search	142	8.0
	Friends or family members (who are veterinary professionals)	129	7.3
	Free vet/nurse hotlines	53	3.0
	Online group(s) (related to my dog’s breed/crossbreed)	52	2.9
	Friends or family members (who are not veterinary professionals)	45	2.5
	Online group(s) (related to my dog’s health condition, if applicable)	29	1.6
	Directly contact breeder	17	1.0
	Directly contact veterinary physiotherapist	17	1.0
	Directly contact animal care staff (e.g., Groomer, dog walker, kennel assistant)	11	0.6
	Miscellaneous	8	0.5
	Online group(s) (general dog related groups)	9	0.5
	Books	3	0.2
	Directly contact pet shop staff	1	0.1
**Top three most frequently used canine health information-sources**	My own existing knowledge/experience	1300	73.7
	Directly contact my local vets/veterinary professionals in practice	1078	61.1
	Internet search	869	49.2
	Friends or family members (who are veterinary professionals)	385	21.8
	Online group(s) (related to my dog’s breed/crossbreed)	281	15.9
	Friends or family members (who are not veterinary professionals)	270	15.3
	Online group(s) (related to my dog’s health condition, if applicable)	255	14.4
	Free vet/nurse hotlines	197	11.2
	Online group(s) (general dog related groups)	147	8.3
	Directly contact breeder	122	6.9
	Directly contact veterinary physiotherapist	81	4.6
	Books	76	4.3
	Directly contact animal care staff (e.g., Groomer, dog walker, kennel assistant)	55	3.1
	Directly contact pet shop staff	7	0.4
	Scientific publications	6	0.3
	TV Shows	5	0.3
	Online group(s)(not related to dogs)	4	0.2
	Podcasts	2	0.1
	Magazines	2	0.1
	Radio	1	0.1
	Telemedicine	1	0.1
	Webinars	1	0.1

First-time owners were more likely to use the internet for researching their dog’s health than experienced dog owners (65.2% vs. 47.0%, respectively: *p* < 0.001) and were less likely to use their own knowledge (59.2% vs. 75.7%, *p* < 0.001) (S5 Table in S1 File). Owners of a dog diagnosed with a long-term condition were more likely to use online dog health related groups than owners without a dog diagnosed with a long-term condition (21.1% vs. 11.7%, *p* < 0.001) (S6 Table in S1 File). There were no significant associations between information-source and owning a brachycephalic purebred or brachycephalic crossbreed (S7 Table in S1 File). Participants reported reputation of the information-source (*n* = 1159, 6.8%) and the information-sources’ factual correctness (*n* = 1138, 65.6%) as the most reported factors that influenced their choice of information-source (Table 4).

**Table 4:** Factors identified by (*n* = 1772) UK dog owners as important when selecting dog health information-sources.

Factors influencing health information-source choice	*n*	%
Reputation	1159	66.8
Factual correctness	1138	65.6
Availability	686	39.5
Accessibility	490	28.2
Ease to understand	417	24.0
Affordability	309	17.8
Miscellaneous	57	3.3

### Vignettes: Accuracy of naming the conditions

**Fig 1:**
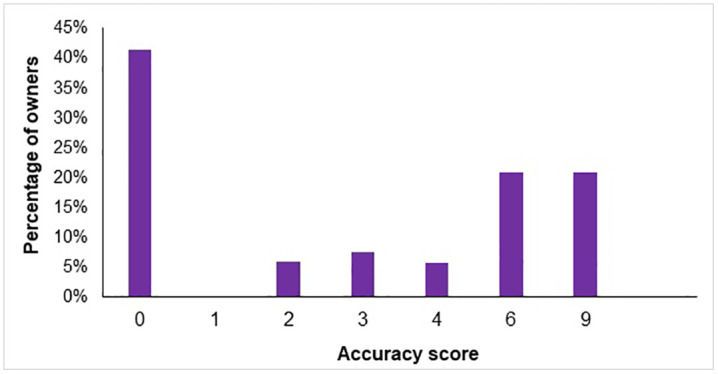
Bar chart of UK dog owners’ (n = 1772) median accuracy scores (0 = least accurate, 9 = most accurate) for identifying common canine conditions featured within vignettes.

Accuracy scores were bimodally distributed with most owners either gaining either very high or very low scores (Fig 1).

**Fig 2:**
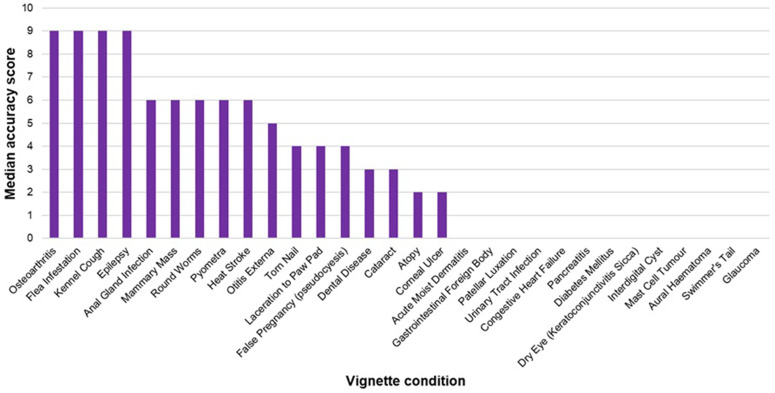
Median accuracy scores of (n = 1772) UK dog owners for identification of common canine conditions (conditions with a median accuracy score of 0 excluded).

Of the 30 conditions featured in the vignettes, epilepsy, flea infestation, kennel cough, and osteoarthritis were the conditions most accurately identified by owners (median score 9/9, IQR 9–9), whereas mast cell tumour, glaucoma and gastrointestinal foreign body were amongst the least accurately identified conditions (median score 0/9, IQR 0–0) (Fig 2).

Accuracy of naming the condition was not significantly associated with the prevalence of the condition (Spearman rho = 0.29, 95% confidence interval (CI) −0.20 – 0.50, *p* = 0.120) or with the condition presentation (i.e., acute, chronic or emergency) (H(3) = 0.27, *p* = 0.873).

Of the 57 predictors tested for association with the vignette condition accuracy, 12 were liberally associated (p < 0.10) and were taken forward for multivariable modelling (S8 and S9 Tables in S1 File). The final multivariable model included ten variables. After accounting for the effects of the other variables in the final model (Table 5), Vignette ID was significantly associated with accuracy. Participants scored 8.15 points lower on accuracy for gastrointestinal foreign body (95% CI −13.4 – −2.9, *p* = 0.002) compared to kennel cough. Owners scored 6.11 points lower on accuracy for congestive heart failure (95% CI −11.4 – −0.9, *p* = 0.023) compared to kennel cough.

**Table 5:** Multivariable generalised linear mixed model for accuracy of UK dog owners (*n =* number of vignette responses) in correctly identifying 30 commonly diagnosed conditions from (*n =* 5316) vignettes.

Variable	Subcategory	B•	Std. Error	*95% CI	*p*-Value
**Intercept**	Intercept	9.26	2.68	4.01 – 14.51	**< 0.001**
**Vignette**	Kennel Cough	REFERENCE			
	**Gastrointestinal Foreign Body**	**−8.15**	**2.67**	**−13.40– − 2.90**	**0.002**
	**Glaucoma**	**−8.11**	**2.67**	**−13.30– − 2.90**	**0.002**
	**Dry Eye (Keratoconjunctivitis Sicca)**	**−8.09**	**2.67**	**−13.30– − 2.80**	**0.003**
	**Mast Cell Tumour**	**−7.93**	**2.67**	**−13.20– − 2.70**	**0.003**
	**Pancreatitis**	**−7.60**	**2.67**	**−12.80– − 2.40**	**0.004**
	**Acute Moist Dermatitis (Hotspot)**	**−7.35**	**2.67**	**−12.60– − 2.10**	**0.006**
	**Patellar Luxation**	**−7.19**	**2.67**	**−12.40– − 1.90**	**0.007**
	**Diabetes Mellitus**	**−7.02**	**2.67**	**−12.30– − 1.80**	**0.009**
	**Atopy**	**−6.94**	**2.68**	**−12.20– − 1.70**	**0.010**
	**Tail Pull/Swimmer’s Tail**	**−6.90**	**2.67**	**−12.10– − 1.70**	**0.010**
	**Interdigital Cyst**	**−6.72**	**2.67**	**−12.00– − 1.50**	**0.012**
	**Corneal Ulcer**	**−6.47**	**2.67**	**−11.70– − 1.20**	**0.016**
	**Aural Haematoma**	**−6.17**	**2.67**	**−11.40– − 0.90**	**0.021**
	**Congestive Heart Failure**	**−6.11**	**2.68**	**−11.40– − 0.90**	**0.023**
	**Dental Disease**	**−5.77**	**2.67**	**−11.00– − 0.50**	**0.031**
	**Urinary Tract Infection**	**−5.58**	**2.67**	**−10.80– − 0.30**	**0.037**
	**False Pregnancy (Pseudocyesis)**	**−5.49**	**2.67**	**−10.70– − 0.30**	**0.040**
	**Cataract**	**−5.18**	**0.35**	**−5.90– − 4.50**	**< 0.001**
	Otitis Externa	−4.74	2.67	−10.00–0.50	0.076
	Laceration To Paw Pad	−4.67	2.67	−9.90–0.60	0.081
	Mammary Mass	−4.29	2.67	−9.50–0.90	0.108
	Anal Gland Infection	−3.89	2.67	−9.10–1.40	0.146
	Torn Nail	−3.64	2.68	−8.90–1.60	0.175
	Pyometra	−3.56	2.67	−8.80–1.70	0.183
	Round Worms	−2.86	2.67	−8.10–2.40	0.285
	Heat Stroke	−2.79	2.67	−8.00–2.40	0.296
	**Epilepsy**	**−1.96**	**0.35**	**−2.60– − 1.30**	**< 0.001**
	Osteoarthritis	−0.92	2.67	−6.20–4.30	0.731
	Flea Infestation	−0.74	2.67	−6.00–4.50	0.781
**Employed Within The Canine Professional Industry**	No	REFERENCE			
	**Yes**	**0.87**	**0.10**	**0.67–1.08**	**< 0.001**
**Owner Age**	18–24 Years Old	REFERENCE			
	Prefer Not To Say	0.72	0.57	−0.39–1.84	0.204
	**75–84 Years Old**	**−1.08**	**0.34**	**−1.74– − 0.42**	**0.001**
	65–74 Years Old	−0.50	0.27	−1.02–0.03	0.064
	**55–64 Years Old**	**−0.54**	**0.26**	**−1.05– − 0.03**	**0.039**
	**45–54 Years Old**	**−0.73**	**0.26**	**−1.24– − 0.23**	**0.005**
	**35–44 Years Old**	**−0.67**	**0.26**	**−1.19– − 0.16**	**0.011**
	**25–34 Years Old**	**−0.60**	**0.27**	**−1.13– − 0.08**	**0.025**
**Information-Source Utilised To Aid Vignette-Based Questions: Internet Search**	No	REFERENCE			
	**Yes**	**0.25**	**0.11**	**0.04–0.46**	**0.018**
**Information-Source Utilised To Aid Vignette-Based Questions: General Dog Groups**	No	REFERENCE			
	**Yes**	**−0.60**	**0.20**	**−1.09– − 0.16**	**0.009**
**Usual Information-Source For Canine Health Information: General Dog Groups**	No	REFERENCE			
	**Yes**	**−0.30**	**0.15**	**−0.58– − 0.01**	**0.041**
**Usual Information-Source For Canine Health Information: Internet Search**	No	REFERENCE			
	Yes	0.11	0.08	−0.06–0.27	0.196
**1st Time Dog Owner**	No	REFERENCE			
	Yes	−0.10	0.13	−0.36–0.15	0.421
**Has A Dog With A Long-Term Health Condition**	No	REFERENCE			
	Yes	−0.09	0.09	−0.27–0.08	0.310
**Has A Brachycephalic Or Brachycephalic Cross Breed**	No	REFERENCE			
	Yes	0.15	0.15	−0.14–0.44	0.303

• Coefficient. *95% confidence interval. Significant values are shown in bold.

Age was also a significant factor, with participants aged 75–84 year olds scoring 1.08 points lower on the vignette accuracy score (95% CI −1.74 – −0.42, *p* = 0.001) compared to 18–24 year olds. Participants that used internet to search for health information to aid their vignette answers scored 0.25 points higher on the vignette accuracy score (95% CI 0.04–0.46, *p* = 0.018) compared to owners who did not. Participants’ that used general online dog groups to aid their vignette answers, scored 0.624 points lower on the vignette accuracy score (95% CI −1.09 – −0.16, *p* = 0.009) compared to owners who did not. Finally, owners that used general online dog groups habitually for canine health information-sourcing for their own dog scored 0.30 points lower on the vignette accuracy score (95% CI −0.58 – −0.01, *p* = 0.042) compared to those that did not.

### Vignettes: Urgency of conditions

**Fig 3:**
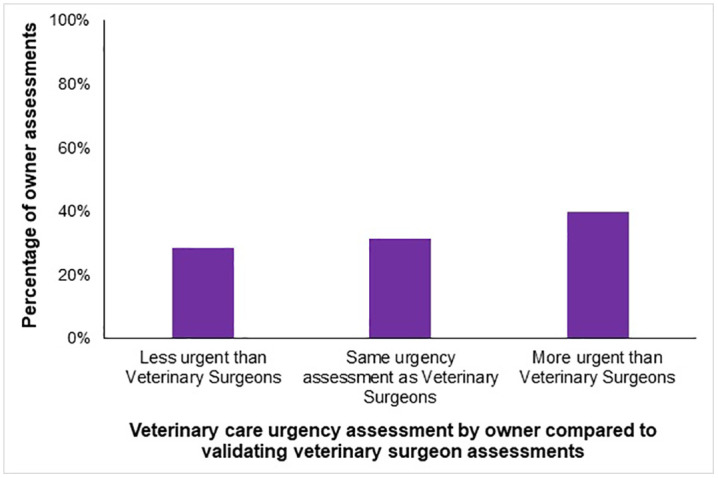
Comparison between (n = 1772) UK dog owners’ assessments of how urgently veterinary care is required for 30 common canine conditions and the recommended timeframes from veterinary surgeons, across n = 5316 assessments of vignettes.

Participants perceived the urgency of the requirement for veterinary care for that case as less urgent than the validating veterinary surgeons in 28.4% (*n* = 1497) of vignettes responses (Fig 3); however, assessments varied widely between individual vignettes.

**Fig 4:**
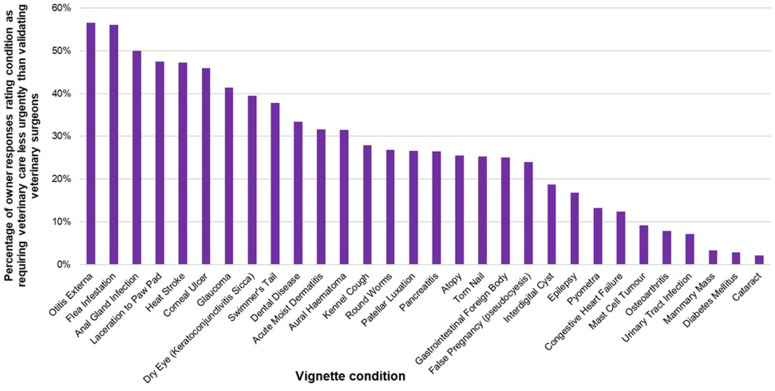
Bar chart comparing percentage of n = 5316 vignette responses from UK dog owners (n = 1772) that identified common canine conditions as requiring veterinary care less urgently than recommended by veterinary surgeons.

Cataracts had the lowest percentage of disagreement in urgency assessment between participants and validating veterinary surgeons with 2.2% (*n* = 3/136) of participants’ assessments underestimating how urgent veterinary care was required compared to veterinary surgeon assessments. In contrast, otitis externa had the greatest percentage of disagreement between participants and veterinary surgeons, with 56.5% (*n* = 166/294) of participants underestimating how urgent veterinary care was required compared to veterinary surgeon assessments (Fig 4).

Owner urgency assessment was not significantly associated with the prevalence of the condition (Spearman rho = 0.184, 95% CI −0.05–0.61, *p* = 0.330).

Of the 57 predictors tested for association with the urgency of seeking veterinary care, 20 were liberally associated and were taken forward for multivariable modelling (S10 Table in S1 File). The final multivariable model retained 13 variables (Table 6). Cataracts was the condition with the greatest level of agreement on urgency for veterinary care needed between owners and veterinary surgeons and thus selected as the reference category for Vignette ID. Within the Vignette ID variable, 24 conditions had significantly different odds to Cataracts for being considered less urgently in need of veterinary attention (Table 6). Otitis externa had 34.13 increased odds of being considered to be less urgent than the validating veterinary surgeons’ assessment compared to cataracts (odds ratios (OR) 34.13, 95% CI 13.04–89.28, *p* < 0.001). Kennel cough had 10.30 increased odds of being considered to be less urgent than the validating veterinary surgeons’ assessment compared to Cataracts (OR 10.30, 95% CI 3.74–28.39, *p* < 0.001). Congestive heart failure had 3.91 increased odds of being considered to be less urgent than the validating veterinary surgeons’ assessment compared to Cataracts (OR 3.91, 95% CI 1.27–12.06, *p* = 0.018).

**Table 6:** Multivariable binary logistic regression model for risk factors for (*n =* 5316) UK dog owners assessing the urgency of veterinary care for (*n =* 30) vignettes as less urgent than veterinary surgeons.

Variable	Subcategory	B•	Std. Error	Odds ratio (OR)	*95% CI	*p*-Value
**Vignette**	1C: Cataract	REFERENCE				
	**7A: Otitis Externa**	**3.53**	**0.49**	**34.13**	**13.04–8 9.28**	**< 0.001**
	**6B: Flea Infestation**	**3.35**	**0.50**	**28.35**	**10.56–76.14**	**< 0.001**
	**5A: Anal Gland Infection**	**3.22**	**0.50**	**24.94**	**9.32–66.74**	**< 0.001**
	**10B: Corneal Ulcer**	**3.20**	**0.51**	**24.60**	**9.11–66.45**	**< 0.001**
	**5B: Laceration to paw pad**	**3.18**	**0.50**	**23.93**	**8.94–64.03**	**< 0.001**
	**8B: Heat Stroke**	**3.11**	**0.50**	**22.38**	**8.38–59.75**	**< 0.001**
	**2C: Dry Eye (Keratoconjunctivitis sicca)**	**2.92**	**0.51**	**18.48**	**6.86–49.76**	**< 0.001**
	**3A: Tail pull/Swimmer’s tail**	**2.81**	**0.49**	**16.66**	**6.32–43.88**	**< 0.001**
	**4B: Glaucoma**	**2.77**	**0.50**	**15.94**	**5.93–42.83**	**< 0.001**
	**7C: Dental Disease**	**2.61**	**0.49**	**13.55**	**5.17–35.53**	**< 0.001**
	**4A: Acute Moist Dermatitis (Hotspot)**	**2.45**	**0.51**	**11.61**	**4.29–31.40**	**< 0.001**
	**10C: Aural Haematoma**	**2.41**	**0.51**	**11.17**	**4.08–30.60**	**< 0.001**
	**1A: Kennel Cough**	**2.33**	**0.52**	**10.30**	**3.74–28.39**	**< 0.001**
	**2A: Patellar Luxation**	**2.27**	**0.51**	**9.70**	**3.56–26.45**	**< 0.001**
	**9A: Atopy**	**2.24**	**0.54**	**9.35**	**3.27–26.76**	**< 0.001**
	**3B: Foreign Body**	**2.18**	**0.50**	**8.84**	**3.32–23.49**	**< 0.001**
	**4C: Round Worms**	**2.17**	**0.51**	**8.75**	**3.22–23.78**	**< 0.001**
	**7B: False Pregnancy (pseudocyesis)**	**2.16**	**0.49**	**8.66**	**3.28–22.83**	**< 0.001**
	**9C: Torn Nail**	**2.16**	**0.55**	**8.64**	**2.92–25.53**	**< 0.001**
	**6A: Pancreatitis**	**2.04**	**0.51**	**7.68**	**2.81–20.97**	**< 0.001**
	**8C: Interdigital Cyst**	**1.80**	**0.52**	**6.07**	**2.21–16.68**	**< 0.001**
	**1B: Epilepsy**	**1.61**	**0.53**	**5.02**	**1.76–14.31**	**0.003**
	**2B: Pyometra**	**1.44**	**0.53**	**4.21**	**1.48–11.95**	**0.007**
	**9B: Congestive Heart Failure**	**1.36**	**0.57**	**3.91**	**1.27–12.06**	**0.018**
	3C: Mast Cell Tumour	1.03	0.53	2.79	1.00–7.82	0.051
	6C: Osteoarthritis	0.79	0.56	2.21	0.74–6.60	0.156
	8A: Urinary Tract Infection	0.71	0.56	2.04	0.68–6.12	0.204
	10A: Mammary Mass	0.37	0.62	1.44	0.43–4.82	0.552
	5C: Diabetes Mellitus	0.11	0.61	1.11	0.33–3.72	0.862
**Information-source used to aid vignette-based questions: Dog health groups**	No	REFERENCE				
	**Yes**	**−0.70**	**0.22**	**0.50**	**0.33–0.76**	**0.001**
**Information-source used to aid vignette-based questions: Would contact veterinary practice despite being asked not to**	No	REFERENCE				
	**Yes**	**−0.92**	**0.45**	**0.40**	**0.17–0.95**	**0.039**
**Usual information-source for canine health information: Contacts veterinary practice**	No	REFERENCE				
	**Yes**	**−0.36**	**0.07**	**0.70**	**0.61–0.81**	**< 0.001**
**Usual information-source for canine health information: Contacts non-veterinary professional friends and family**	No	REFERENCE				
	**Yes**	**0.33**	**0.10**	**1.39**	**1.15–1.68**	**< 0.001**
**Usual information-source for canine health information: General dog groups**	No	REFERENCE				
	**Yes**	**0.26**	**0.13**	**1.30**	**1.01–1.66**	**0.040**
**Regularly uses veterinary prescription only flea and worm prevention**	No	REFERENCE				
	**Yes**	**−0.47**	**0.07**	**0.63**	**0.54–0.72**	**< 0.001**
**Currently insured**	No	REFERENCE				
	**Yes**	**−0.23**	**0.07**	**0.80**	**0.69–0.92**	**0.003**
**Information-source utilised to aid vignette-based questions: Internet search**	No	REFERENCE				
	Yes	−0.11	0.10	0.90	0.74–1.09	0.261
**Usual information-source for canine health information: Internet search**	No	REFERENCE				
	Yes	0.07	0.07	1.07	0.92–1.24	0.374
**1st time dog owner**	No	REFERENCE				
	Yes	0.00	0.11	1.00	0.80–1.25	0.988
**Has a dog with a long-term health condition**	No	REFERENCE				
	Yes	−0.10	0.08	0.91	0.78–1.07	0.240
**Has a brachy-cephalic or brachy-cephalic cross breed**	No	REFERENCE				
	Yes	−0.01	0.13	0.99	0.76–1.29	0.939

• Coefficient. *95% confidence interval. Significant values are shown in bold.

Owners that searched for information using online dog health groups had 0.50 decreased odds (95% CI 0.33–0.76, *p* = 0.001) of considering vignette scenarios to be less urgent than the validating veterinary surgeons’ assessment compared with owners that did not. Owners who would have contacted their veterinary practice to assist them with their vignette responses had 0.40 decreased odds (95% CI 0.17–0.95, *p* = 0.039) of considering vignette scenarios to be less urgent than the validating veterinary surgeons’ assessment compared with those that would not. Owners that would usually contact their veterinary practice for canine health information had 0.70 decreased odds (95% CI 0.61–0.81, *p* < 0.001) of considering vignettes scenarios to be less urgent than the validating veterinary surgeons’ assessment compared with those that would not. Owners that usually used online general dog groups for canine health information had 1.30 increased odds (95% CI 1.01–1.66, *p* = 0.040) of considering vignette scenarios to be less urgent than the validating veterinary surgeons’ assessment compared with owners who did not.

### Comparison of accuracy and urgency

**Fig 5:**
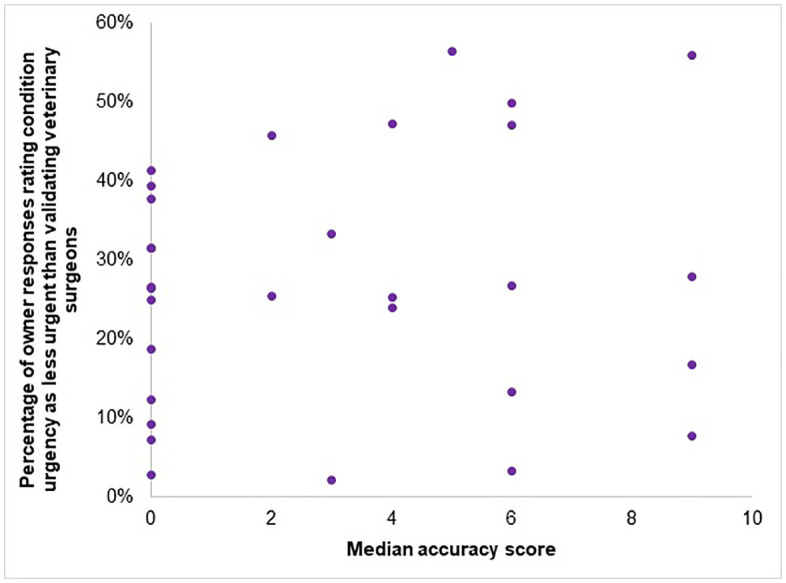
Scatter plot to compare UK dog owners’ median accuracy of condition identification in n = 5316 vignette responses with average percentage of owners that perceived the requirement of veterinary care to be less urgent than recommendations from veterinary surgeons.

There was no correlation between owners’ accuracy of condition identification and their assessments of urgency for veterinary care (Spearman rho = 0.13, 95% CI −0.24–0.46, *p* = 0.482) (Fig 5).

## Discussion

This study used a novel vignette-based approach to explore key aspects of dog owners’ knowledge of canine health and decision-making regarding seeking veterinary care. The study specifically evaluated owner ability to identify conditions based upon observed changes to a dog’s physical condition and/or mental demeanour, and the timeframe within which they would seek veterinary care for each condition. Results demonstrated great heterogeneity in responses based upon the condition presented within vignettes; for those conditions with very clear external clinical signs (e.g., kennel cough), the results reassuringly suggest that dog owners generally have good awareness and are accurate at identifying appropriate diagnoses. However, for many conditions that show vague clinical signs, owners were often highly inaccurate in their identification. Worryingly, a large proportion of owners perceived many of the disorders featured to require veterinary care less urgently than was considered appropriate by a group of experienced small animal veterinary surgeons. This raises concerns for negative canine welfare because of non-, under- or delayed treatment.

### Accuracy of condition identification

Of the 30 disorders examined, epilepsy, kennel cough, flea infestation and osteoarthritis were the most accurately identified conditions by owners. While only veterinary surgeons can legally diagnose conditions [6], owners’ ability to recognise conditions remains vital to animal welfare. The conditions that owners accurately identified in the current study could be considered generally easier to recognise and are often diagnosed by a veterinary surgeon from a physical examination alone from their clear, external clinical signs (e.g., the characteristic cough of kennel cough, and visible external parasites for flea infestation) [65]. In contrast, the conditions least accurately identified by owners tended to show variable, unclear presentations such as for mast cell tumours, glaucoma and gastrointestinal foreign bodies. These least accurately identified conditions could be considered as more complex and generally are challenging to diagnose by veterinary surgeons from physical examination alone, often requiring additional diagnostic testing. Definitive biomedical diagnoses are reached in as few as 20.7% of small-animal consultations [65], yet appropriate treatment is still often possible within contextualised care [8].

The vignettes in the current study offered stereotypical presentations of each condition to facilitate easier comprehension by participants without setting owners up for failure by providing atypical clinical signs [66]. However, because real-world presentations of the conditions covered in the vignettes can vary greatly [65], it may be that the results from the current study can only fully generalise to ‘typical’ presentations of the disorders studied. A key example in this study is osteoarthritis, a condition that was generally well identified by owners in the current study. As a prevalent, age-associated condition [67,68], osteoarthritis can manifest in subtle behavioural changes that precede visible lameness [69]. While owners recognised the typical vignette presentation, both owners and veterinary surgeons often report uncertainty in recognising atypical manifestations [70].

The current study demonstrated that owner accuracy of condition identification increases with scenario-specific internet searching, and thus the use of online information-prescriptions by veterinary professionals could be valuable for conditions where accuracy was found to be low. Information-prescriptions can direct owners to relevant and reliable information, allowing owners the opportunity for autonomy in their dog health research [71]. How and when the public are accessing and using the internet and online resources is rapidly evolving [72] and whilst veterinary-recommended information prescriptions are a potential benefit to owners and for animal welfare, growing concerns have been expressed over misinformation for pet owners using internet searches [14,73,74]. Veterinary signposting to curated, high-quality online resources can help owners extend their understanding outside the consultation while maintaining information quality [75]. Greater uptake by veterinary surgeons and owners of information prescriptions within an overall framework of planned veterinary care could facilitate better online searching by clients and offer positive impacts for both animal health and client satisfaction [71]. Despite owners generally welcoming information prescriptions from their veterinary surgeons [76], owner compliance with recommended veterinary care is complex and has been reported as an ongoing challenge for the profession over many years [77]. Increasing adoption of contextualised care [8] and prioritising patient-client-veterinary relationships are key to ensuring information-prescriptions are appropriate for each individual owner [37,76].

In addition to general internet searches for health information, owners also report online dog groups as an important source of canine health related information [55]. Although owners who used the internet to search for health information to aid their vignette answers scored significantly higher on the vignette accuracy score, owners who used more general online dog groups (i.e., dog sport groups), whether habitually for their own dog’s health or specifically to aid their vignette responses, were less accurate at identifying the vignette conditions. Online discussion groups often involve lay individuals offering each other advice regarding their dogs’ health, wellbeing and general aspects of dog ownership are unregulated and do not have moderation by veterinary professionals [55]. Somewhat paradoxically despite their common use, many owners do not regard Facebook groups as a trustworthy canine health information-source [55]. The current results suggest that some online groups could be a cause for concern for the veterinary profession due to reliance by owners on interpretations by other lay persons of their dog’s clinical signs which could result in ‘misdiagnosis’ and inappropriate or delayed treatment. Professionally moderated forums and veterinary-led tools such as PetsApp [78], VidiVet [79] and CAM [80] demonstrate that credible, accessible online support is achievable and could be expanded to other common chronic conditions [81].

### Urgency assessments

From a welfare perspective, the ability of owners to recognise when veterinary care is required may be more important than accurate recognition of the precise condition, which remains the remit of veterinary surgeons [6]. Veterinary examination can identify clinical signs that owners may miss [82]. Consequently, it is concerning that over a quarter of owners underestimated the urgency of veterinary care recommended by veterinary surgeons in the vignettes. Whilst some owners overestimated the urgency of the vignettes relative to veterinary surgeons’ assessments, underestimation is arguably a greater concern for canine welfare; however, patient triage and workflow effectiveness may be hampered by owners seeking care prematurely. Although these vignettes were hypothetical, they were based upon real clinical histories and stereotypical presentations from VetCompass [57].

Despite vignette-specific internet searching improving owners’ condition identification, internet searching (both habitually and vignette-specific) was not associated with seeking veterinary care in an appropriate timeframe. Owners who habitually used general online dog groups (e.g., breed-specific groups, dog sport groups) for canine health information were less likely to seek veterinary care in an appropriate timeframe, whereas those that used online dog health-related groups to aid their vignette answers were more likely to consider conditions to require veterinary care in an appropriate timeframe. These contrasting results for online dog-related groups highlight potential differences in the user-generated content and the prevailing culture within online communities that may influence owners’ health literacy and perceptions of urgency for different conditions. The level of engagement by owners for information-sourcing from online groups may vary widely, from active participation and advice-seeking to passive observation of others’ experience [55]. In the context of the current study, dog health discussions amongst owners in online groups (whether disease specific groups, or more general) appear to have varying effects on the timescales for seeking veterinary care. Further study is warranted to understand why these differences occur, including qualitative analysis of online discussions [42,83], as has been recently explored in other canine contexts [84,85].

Otitis externa was the condition with the greatest percentage of owners considering veterinary care to be less urgently required than the validating veterinary surgeons’ recommendations. Otitis externa is a common, painful condition where the lining of the dog’s ear(s) becomes inflamed and thickened [86]. Delayed treatment or non-treatment can increase the welfare cost by increasing duration and severity [63,86]. The urgency of presentation for heatstroke was similarly underestimated by owners, although heatstroke can be rapidly fatal in dogs, so a high level of owner understanding of the urgency for seeking veterinary care is critical for this condition [87]. Improving owner education around the importance of timely access to veterinary care for emergency conditions such as heatstroke in dogs could be done through national information campaigns. It is thus of concern that high profile public information campaigns such as ‘dogs die in hot cars’ [88] and ‘dogs die on hot walks’ [87,89,90] are not sufficiently raising awareness amongst the public around the urgency of presenting a dog affected by heatstroke to a veterinary surgeon, even if they have had success regarding educating owners on which contexts to avoid that could precipitate heatstroke.

Owners who reported they would have contacted their local veterinary practice as part of their vignette answers and those owners who habitually contact their veterinary practice for canine health information/advice in the real world were more likely to seek veterinary care in appropriate timeframes. This supports previous evidence that regular engagement with veterinary teams is associated with more welfare-appropriate decision-making [76,91]. In addition to veterinary surgeons and nurses, veterinary reception staff are generally well trained in triage, prioritisation of cases and advising owners on the urgency that veterinary care is needed [92]. Veterinary teams represent a key touchpoint in urgency assessment, but, deploying their advice relies on owners acknowledging the practice as a trusted and accessible source of support.

In contrast to seeking advice from veterinary professionals, those owners in the current study who sought non-veterinary opinions, e.g., those that usually contact friends and family for canine health information/advice (not including veterinary professionals), were less likely to seek veterinary care in an appropriate timeframe. This reinforces the importance of veterinary expertise over lay interpretations when assessing canine health conditions [93]. Promoting owner engagement through interactive practice resources could further encourage timely veterinary attendance [94].

Telephone triage services provide concerned owners with timely reassurance regarding their pet’s health, offering a more reliable alternative to unverified online often termed “Dr Google.” Many owners lack the knowledge to identify credible online information or use appropriate search terms, which can lead to confusion or misinformed decisions. The BVA considers consultation with a local veterinary practice, including thorough telephone triage, the “safest” approach with respect to animal welfare and recommends this over unsupervised internet searches for health information [95]. However, while broad public messaging encouraging owners to engage directly with their veterinary practice is valuable, its implementation must consider the existing workload pressures on an already stretched veterinary workforce [96]. Applications such as PetsApp [78] and VidiVet [79] offer accessible digital triage solutions at little or no cost to owners and can support practices by helping to prioritise unwell patients more effectively. Similarly, free veterinary helplines offered through some insurance providers [97] were utilised by only 11.2% of dog owners in the present study, yet such services have potential to enhance owner decision-making while alleviating the workload of frontline veterinary teams. Increasing the availability and visibility of these professional support options could reduce the likelihood of owners seeking non-professional or unreliable online advice, thereby safeguarding animal welfare.

Although this study was conducted prior to the widespread availability of AITs, such technologies are now increasingly influencing how individuals seek and interpret health information. Although data on use in companion animal owners is currently not available, recent human healthcare studies have shown that members of the public are using conversational AITs, such as ChatGPT, for self-diagnosis and health-related queries [98], with concerns raised about their potential to generate inaccurate or misleading information without appropriate professional oversight [99]. Integrating AIT-driven advisory systems within structured, professionally supervised triage frameworks could therefore represent both an opportunity and a risk for veterinary healthcare delivery, underscoring the continued importance of clinician validation and guidance.

### Influences on owner accuracy and urgency assessments

Several owner-specific factors were associated with their ability to assess condition accuracy and urgency appropriately. Younger owners (18–24 years old) were the most accurate at identifying conditions, possibly reflecting their greater familiarity with online information sourcing [100]. First-time owners were more likely to use the internet for researching their dog’s health than experienced dog owners and were less likely to rely on their own knowledge, which may represent a welfare-positive behaviour if guided appropriately. Although some veterinary professionals’ express concerns about owners replacing veterinary advice with internet searches [14], signposting and educational activities such as information guides or puppy classes can help new owners access reliable information and foster positive relationships with veterinary teams [101–103].

No associations were identified between ownership of a dog diagnosed with a long-term condition and either the accuracy of vignette condition identification or appropriate urgency assessments. However, these owners showed greater habitual use of online dog-health groups, consistent with previous findings that carers of chronically ill pets may seek frequent emotional and informational support online [54,104]. Professionally moderated forums may therefore represent a useful model for providing accessible, day-to-day support these individuals.

Although there is a large body of evidence supporting substantially poorer overall health and longevity in brachycephalic dogs compared with non-brachycephalic dogs [105–107], the current study found no associations between brachycephalic ownership and either condition identification or urgency assessments. Given the increasing prevalence of these compromised breeds and known perceptual biases among their owners [41,105], further research is warranted in this area.

### Preferred dog health information-sources

The reputation of the information-source and its factual correctness were the most important factors that influenced dog owners’ choice of information-source. To maximise factual correctness, owners would be well-placed to seek advice from veterinary professionals directly, or veterinary created/endorsed resources. However, reputation of veterinary surgeons with the general public appears low at present in the UK, with recent qualitative data finding that some UK veterinary surgeons feel that some clients don’t fundamentally trust the profession, with clients’ perceptions of veterinary commercial interests contributing to this [29]. This may partly be a reflection of the rising costs of veterinary care during the cost-of-living crisis, that are above inflation [108]. Media portrayals of veterinarians as ‘profit-hungry’ [109] may have exacerbated public distrust, although affordability ranked low among owner priorities. Online berating or abuse of veterinary professionals or practices could be a reflection in the breakdown in this relationship [35], with recent reports that 48% of veterinary surgeons have been verbally abused online. In response, the British Veterinary Association (BVA) have launched a campaign to ‘end the abuse of veterinary professionals’ [110]. Implementation of more open discussions regarding the financial elements of all potential diagnostic and treatment approaches for disorders, utilising shared-decision-making strategies between veterinary surgeons and owners could help to improve owners’ experience satisfaction and improve their perception of value for money [111], and improve the reputation of the profession as a trusted source of animal health advice and care. Open discussions regarding information sourcing may further improve veterinary surgeon-owner relationships, with a previous study reporting that veterinary surgeons asking owners about their animal health information-sourcing and actively making recommendations for good information-sources was positively associated with shared-decision-making [91].

### Study limitations and areas for future study

Although this study benefited from a relatively large sample, due to the largely unknown demographics of the UK dog owning population (e.g., due to no ownership licensing system being in place), the sample cannot be considered representative of all UK dog owners and may be biased towards those with a greater interest in dog health given participants were self-selected. Results also relied upon owners self-reporting what sources they used for assisting vignette responses, which may not be honest or accurate. Online ‘Proctoring’, which can be an online form of invigilating [112], could be a means of directly observing owners’ behaviours around information-sourcing, but setting up a study involving this would require extensive planning, consent and resources. The current study offered hypothetical cases, that albeit based upon real clinical histories, provided only a limited context for each case. A next step following these findings could be to move this research question into a fully real-world setting, following owners from when their dog first presents with perceived signs of illness and monitoring their health information-sourcing and proclivity to seek veterinary care over the course of their dog’s condition. Since the data were collected for this study, the development of AITs for public use presents an opportunity to explore owner information-seeking behaviours and decision-making assisted by these technologies, which should be a priority for future research in this area.

## Conclusions

The current findings suggest that UK dog owners’ abilities to accurately identify conditions and their decision-making when determining their urgency for seeking veterinary care are varied and often poor, particularly for complex conditions without clear external physical signs. Although some owners were very good at identifying some conditions, a large proportion underestimated the timeframe in which veterinary care should be sought, contradicting veterinary surgeons’ assessments, which was not related to their ability to identify the condition the hypothetical dog was affected by. This implies owner ability to specifically identify what is wrong with a dog is not crucial in making an appropriate judgement on the urgency for veterinary care, and vice versa. Consequently, owner educational initiatives should focus on alerting owners to key clinical signs that require urgent veterinary care, with opportunities for veterinary professions to make enhanced use of information-prescriptions to support better online information-sourcing by owners. Given current resourcing constraints in veterinary practice, and the negative impact of the cost-of-living crisis on owner finances, triage services and telemedicine could offer owners more ready access to reliable, bespoke advice from the veterinary team and enable frontline veterinary staff to prioritise their workload whilst protecting canine welfare. Although this study predates the widespread emergence of AITs, such technologies have the potential to further shape owner information-sourcing and decision-making behaviours in the future, highlighting the importance of professional oversight to ensure their safe and accurate use within veterinary healthcare.

## Supporting information

S1 File Study advertisement poster, full survey text, clinical vignettes, example accuracy scoring, responses per vignette, univariable analyses. (DOCX)
