# Supplementary material for: To see or not to see the vet: A vignette-based study of decision-making by UK dog owners regarding seeking veterinary care for commonly presenting conditions
Source: PLoS One. 2026 Jan 16;21(1):e0339723. doi: 10.1371/journal.pone.0339723 (PMC12810856; doi:10.1371/journal.pone.0339723)
Supplement: S1 File — (DOCX) [file pone.0339723.s001.docx]

**S1 Figure: Example participant recruitment poster.**


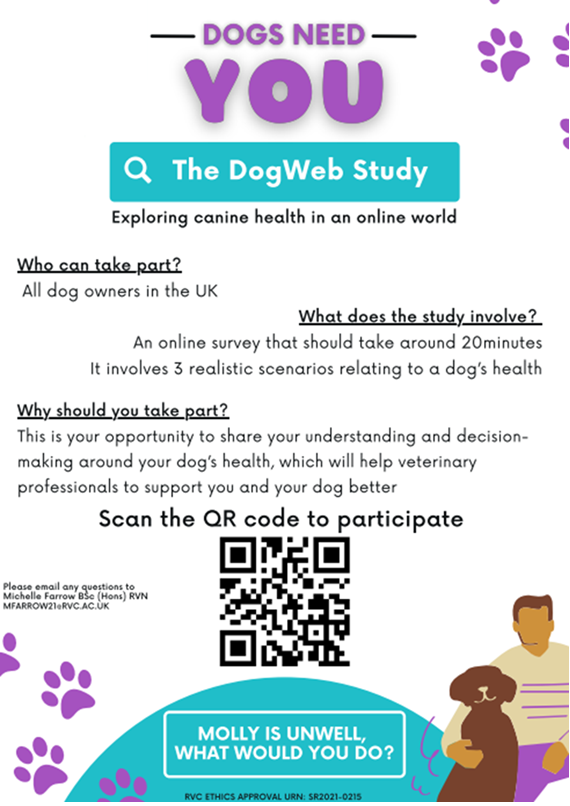


**S2 Text: Full Survey text**

**The DogWeb Study**

Canine healthcare in an online world

1. **What is this survey about?**

We are a team of researchers at the Royal Veterinary College (RVC) who want to improve the lives of dogs in the UK, but we need your help!

Dog ownership can be a rewarding but emotional time for owners, particularly when our canine companions become unwell. We are interested in learning **how** you, a dog owner in the UK, recognise and assess the urgency of treatment required by some common conditions in dogs. This is your opportunity to help us understand your decision-making around your dog’s health, which will help veterinary professionals to support you and your dog better. This will help to improve the relationship between you and your veterinary professionals so we can work towards a mutual goal of optimising canine welfare.

1. **Who can take part?**

We would like to hear from dog caregivers who meet **all** of the following three criteria:

1. Are over 18 years of age.
2. Resident in the United Kingdom.
3. Currently have one or more dogs.

*N.B. Unfortunately, if you do not have a dog currently, on this occasion we ask that you do not participate as some of our questions focus on your dog’s health at present.*

We ask for only one response per household.

1. **What does the study involve?**

To take part, you simply need to answer a series of questions about your approach to healthcare in dogs online. We think you will find the survey interesting and thought-provoking. The whole survey should take approximately **20 minutes.**

The survey will be open until MIDNIGHT 31^ST^ MARCH. Please note the survey end date may vary depending on the amount of participation the survey received.

This survey is to explore how UK dog caregivers approach recognising and deciding on how to manage some common conditions in dogs. You will be presented with **3 SCENARIOS** in which a dog is showing physical or mental changes suggestive of a potential health problem. We ask that you work through these scenarios in your own time – you will be free to research your answers as if you were the caregiver for that dog e.g. speaking with a friend or family member, accessing the internet, reading from a book.

We would like you to imagine that, in each scenario, you are the caregiver for that dog, and are therefore responsible for their health. Please gather as much information as you would for your own dog and answer the questions to the best of your ability. For each scenario, you will be asked to suggest the condition that you feel the dog may be affected by, suggest if and how urgently you feel that dog needs veterinary attention, and also think about how you might feel if your dog was showing the same physical or mental changes as the dog in the scenario. Please note that this is not a test and that only qualified veterinary surgeons are legally allowed to diagnose conditions in animals. After the scenarios, we will ask you for a little information about you and your own dog, all of which will be kept anonymous.

Once you have started the survey, you will need to answer the questions from the same device (e.g. smartphone, PC) in one go. Once you submit the survey to us, you will no longer be able to access your responses.

Completion of the entire survey takes around 20 minutes of your time, but can contribute hugely to a lifetime of improved welfare for dogs in the future.

1. **What will happen to my information?**

Your information will be kept by the RVC in accordance with data protection legislation (GDPR). Only the scientists undertaking this study (Dr. Rowena Packer, Dr. Dan O’Neill, Michelle Farrow, and team) will have access to the originally submitted data. The dataset will be anonymised for research and only anonymised results will be published. Publications may include quotes from the free text responses, but these will be anonymised and you will not be identifiable from any quotes. This study received ethical approval from RVC Social Science Ethical Review Board (URN: SR2021-0215).

1. **Can I change my mind and withdraw my data?**

You are free to withdraw your data from the study until it enters our analysis on 1^st^ April 2022, after which it will be fully anonymised and we will be unable to identify your response to remove it. If you would like to withdraw your response before 1^st^ April 2022, please contact Michelle Farrow (mfarrow21@rvc.ac.uk). Any data that you request to be withdrawn will be permanently deleted.

1. **What if taking part raises concerns?**

If this study raises any concerns about your dog's welfare, then please contact a veterinary surgeon to discuss. You may find a local veterinary surgeon at <https://findavet.rcvs.org.uk/home/>

Resources on specific canine welfare issues raised will also be included at the end of the survey.

If this study raises any concerns about your own mental health, then please access this NHS resource:

[www.nhs.uk/conditions/stress-anxiety-depression/mental-health-helplines/](http://www.nhs.uk/conditions/stress-anxiety-depression/mental-health-helplines/)

1. **CONSENT**(Tick box statement)

I confirm that I have read and understood the above information and give consent for my answers to be used for this research study and any resulting publications

If you have any further questions about the study, please contact the primary study investigator, Michelle Farrow ([mfarrow21@rvc.ac.uk](mailto:mfarrow21@rvc.ac.uk)).

1. **INCLUSION CRITERIA**
2. Are you 18 years old or over?

YES or NO

1. Are you a UK resident?

YES or No

1. Do you currently have at least one dog?

YES or NO

**IF NO TO ANY OF THE INCLUSION CRITERIA SEND TO EXCLUSION PAGE:** Thank you for your interest in participating in this survey. Unfortunately, you do not meet the criteria for completing this survey.

1. **CASE SCENARIOS**

**For each scenario, please read the description of the dog’s condition and information about the dog. We ask that you then suggest the most likely type of condition you think the dog may be affected by; how urgently you feel the dog in the scenario requires veterinary care (if at all); the most likely part of the body affected; how anxious (worried) you would feel if your dog was showing the same physical or mental changes as the dog in the scenario and how confident you are in your answers to that scenario. *- You are free to access additional resources to assist your answers to these questions in any way, although we kindly request that you do NOT contact your local vets for these scenarios. Please note this is not a test; we are interested in learning about your typical approach to healthcare in dogs.***

***Please read the following scenario and imagine that you are the caregiver for this dog***

***[Insert scenario 1 text here as an example]***

1. **Most likely type of condition** – Please suggest the most likely type of condition you believe this dog may be affected by.

[FREE TEXT] Please note this is not a test and there is not particular correct answer based on this scenario. If you are not sure, please say ‘I am not sure’.

1. **Urgency of condition** –: How soon do you feel you should seek veterinary advice and care, if at all, for this dog? Please select which you feel is the most appropriate option below
   - - - 1. seek veterinary advice and care immediately (within an hour)
         2. seek veterinary advice and care within 12 hours
         3. seek veterinary advice and care within 24 hours
         4. seek veterinary advice and care within a week (7 days)
         5. seek veterinary advice and care within a fortnight (two weeks – 14 days)
         6. self-care (don’t seek veterinary advice and care immediately, try to manage the condition at home)
2. **Most likely part of the body affected–** Please select for each case the body system you feel is most likely to be affected.

- integumentary (skin)
- skeletal (bones)
- muscular (muscles)
- lymphatic (immune system)
- respiratory (airways and lungs)
- digestive (teeth, gut)
- nervous (nerves, spinal cord and brain)
- endocrine (hormones)
- cardiovascular (heart and blood)
- urinary (kidneys, bladder and urethra)
- reproductive (penis/testes/prostate or vagina/ovaries/uterus)

1. **How anxious (worried) you would feel if the dog in the scenario were your dog** – Please score how anxious (worried) you would you feel if it was your current dog in this scenario:

SCALE 1 (Not at all anxious/worried), 2 (not very anxious/worried), 3 (neutral), 4 (fairly anxious/worried), 5 (very anxious/worried)

1. **How confident you are in in the accuracy of your responses –** Please score how confident you are in your answers for this scenario:

SCALE 1 (Not at all confident), 2 (not very confident), 3 (neutral), 4 (fairly confident), 5 (very confident)

**Vignette examples:**

| **Number** | **Scenario** | **Breed, age, sex, neutered or entire** | **Please suggest the most likely type of condition you believe this dog may be affected by** | **How urgent do you feel this condition is?** | **Which part of the body is most likely affected?**  Please select from list | **How anxious (worried) would you be if this was your dog?**  1 (Not at all anxious/worried), 2 (not very anxious/worried), 3 (neutral), 4 (fairly anxious/worried), 5 (very anxious/worried), | **How confident are you in your responses for this scenario?**  (Not at all confident), 2 (not very confident), 3 (neutral), 4 (fairly confident), 5 (very confident), |
| --- | --- | --- | --- | --- | --- | --- | --- |
| 1. | Rosie went to an event over the weekend and there were lots of dogs interacting. She has now developed cough (gag/sneeze/retch) and now seems more tired than usual. It is midday on Tuesday. | “Rosie”  Is a 5 year old neutered female, West Highland White Terrier |  | a. seek veterinary advice and care immediately (within an hour)  b. seek veterinary advice and care within 12 hours  c. seek veterinary advice and care within 24 hours  d. seek veterinary advice and care within a week (7 days)  e. seek veterinary advice and care within a fortnight (two weeks – 14 days)  f. self-care (don’t seek veterinary advice and care immediately, try to manage the condition at home) | • integumentary (skin)  • skeletal (bones)  • muscular (muscles)  • lymphatic (immune system)  • respiratory (airways and lungs)  • digestive (gut)  • nervous (nerves, spinal cord and brain)  • endocrine (hormones)  • cardiovascular (heart and blood)  • urinary (kidneys, bladder and urethra)  • reproductive (penis/testes/prostate or vagina/ovaries/uterus) | 1  2  3  4  5 | 1  2  3  4  5 |
| 2. | George has been on and off skipping/limping on the right hind leg during his walk this morning. This is the first time he has ever done this. It is 9am on a Friday. | “George”  Is a 6month old entire, male Maltipoo (Maltese cross Poodle) |  | a. seek veterinary advice and care immediately (within an hour)  b. seek veterinary advice and care within 12 hours  c. seek veterinary advice and care within 24 hours  d. seek veterinary advice and care within a week (7 days)  e. seek veterinary advice and care within a fortnight (two weeks – 14 days)  f. self-care (don’t seek veterinary advice and care immediately, try to manage the condition at home) | • integumentary (skin)  • skeletal (bones)  • muscular (muscles)  • lymphatic (immune system)  • respiratory (airways and lungs)  • digestive (gut)  • nervous (nerves, spinal cord and brain)  • endocrine (hormones)  • cardiovascular (heart and blood)  • urinary (kidneys, bladder and urethra)  • reproductive (penis/testes/prostate or vagina/ovaries/uterus) | 1  2  3  4  5 | 1  2  3  4  5 |
| 3. | Holly had an unusual episode whilst at home where she fell to the floor, became unresponsive, and was foaming at mouth. She remained unresponsive and her legs were paddling (twitching/kicking legs) for around 60seconds. She then stood up and was appeared to be back to normal within 5 minutes. She had never done this before. It is 9pm on a Tuesday evening. | “Holly”  Is a 1 year old, entire female Border Collie |  | a. seek veterinary advice and care immediately (within an hour)  b. seek veterinary advice and care within 12 hours  c. seek veterinary advice and care within 24 hours  d. seek veterinary advice and care within a week (7 days)  e. seek veterinary advice and care within a fortnight (two weeks – 14 days)  f. self-care (don’t seek veterinary advice and care immediately, try to manage the condition at home) | • integumentary (skin)  • skeletal (bones)  • muscular (muscles)  • lymphatic (immune system)  • respiratory (airways and lungs)  • digestive (gut)  • nervous (nerves, spinal cord and brain)  • endocrine (hormones)  • cardiovascular (heart and blood)  • urinary (kidneys, bladder and urethra)  • reproductive (penis/testes/prostate or vagina/ovaries/uterus) cardiovascular (heart/blood)  urinary  reproductive | 1  2  3  4  5 | 1  2  3  4  5 |

1. **HOW DID YOU REACH YOUR ANSWERS FOR EACH SCENARIO? NB answer selections randomised**
2. Please select the one source that was **most influential** in your **decision making** for this scenario

My own existing knowledge

Internet search

Books

Directly contacted Pet care staff

Directly contacted Veterinary physiotherapist

Directly contacted Breeder

Podcasts

Magazines

TV shows

Radio

Friends or family members (who are not veterinary professionals)

Friends or family members (who are veterinary professionals)

Online group(s) (related to my dogs breed/crossbreed)

Online group(s) (related to my dogs health condition, if applicable)

Online group(s) (general dog related groups)

Online group(s) (not related to dogs)

Other (please specify)

1. What sources of new information did you actively seek out or use while considering your answers for this scenario? Please select all that apply

My own existing knowledge

Internet search

Books

Directly contacted pet care staff

Directly contacted veterinary physiotherapist

Directly contacted Breeder

Podcasts

Magazines

TV shows

Radio

Friends or family members (who are not veterinary professionals)

Friends or family members (who are veterinary professionals)

Online group(s) (related to my dogs breed/crossbreed)

Online group(s) (related to my dogs health condition, if applicable)

Online group(s) (general dog related groups)

Online group(s) (not related to dogs)

Other (please specify)

1. **Logic: If answer to 2. not internet search next section, If internet search continue**

Once you were on your preferred search engine (e.g. google), in your own words, please briefly describe what you did to help you decide on your answers for this scenario.

Free Text

1. Did your internet search influence the answer you selected for how urgently you would seek veterinary advice and care for that dog?

Yes – I decided to seek veterinary care and advice sooner after an internet search

Yes – I decided to seek veterinary care and advice later after an internet search

No – My internet search did not influence my answer for when to seek veterinary care and advice

Other (Please specify)

I don’t know

If you would like to explain your answer please do so here

1. How did your **anxiety** for the **welfare of the dog** in the scenario compare before and after your internet search?

I was more anxious before my internet search and then less anxious after my internet search

I was less anxious before my internet search and then more anxious after my internet search

My level of anxiety was about the same before and after my internet search

Other (Please specify)

I’m not sure

If you would like to explain your answer please do so here

1. How did your **confidence** in in the **accuracy of your responses** for the scenarios compare before and after your internet search?

I was more confident before my internet search and then less confident after my internet search

I was less confident before my internet search and then more confident after my internet search

My confidence was about the same before and after my internet search

Other (Please specify)

I’m not sure

If you would like to explain your answer please do so here

1. **When you have a concern about your dog’s health and are trying to decide whether to seek veterinary care, what do you usually do? NB answer selections will be randomised**
2. When you have a concern about your dog’s health and are deciding whether to seek veterinary care, which **three sources** do you **most frequently** seek out or use?

My own existing knowledge

Contact my local vets

Internet search

Books

Veterinary professionals in practice

Free vet/nurse hotlines

Directly contacted Pet care staff

Directly contacted Veterinary physiotherapist

Directly contacted Breeder

Podcasts

Magazines

TV shows

Radio

Friends or family members (who are not veterinary professionals)

Friends or family members (who are veterinary professionals)

Online group(s) (related to my dogs breed/crossbreed)

Online group(s) (related to my dogs health condition, if applicable)

Online group(s) (general dog related groups)

Online group(s) (not related to dogs)

Other (please specify)

1. When you have a concern about your dog’s health and are deciding whether to seek veterinary care, which **one source** is **most influential** in your decision-making?

My own existing knowledge

Contact my local vets

Internet search

Books

Veterinary professionals in practice

Free vet/nurse hotlines

Directly contacted Pet care staff

Directly contacted Veterinary physiotherapist

Directly contacted Breeder

Podcasts

Magazines

TV shows

Radio

Friends or family members (who are not veterinary professionals)

Friends or family members (who are veterinary professionals)

Online group(s) (related to my dogs breed/crossbreed)

Online group(s) (related to my dogs health condition, if applicable)

Online group(s) (general dog related groups)

Online group(s) (not related to dogs)

Other (please specify)

1. When you have a concern about your dog’s health and are considering whether to seek veterinary care, what are the most important factors that influence where and how you seek out information to help you? Please select all that apply

Affordability

Factual correctness

Ease to access

Ease to understand

Availability

Other (Please specify)

**START NEW PAGE: Searching the internet to help make decisions about dog health**

1. if you have previously had a concern relating to your dog’s health and were deciding whether to seek veterinary care, have you have ever searched the internet for information to help make your decision?

**Yes**

**No**

1. **LOGIC IF YES** Once you were on your preferred search engine (e.g. google), in your own words, please briefly describe what you did to help you decide whether to seek veterinary care

Free Text

**LOGIC IF NO ONTO QU6**

**START NEW PAGE: Conversations with professionals at your veterinary practice**

1. Have you spoken to professionals at your veterinary practice about searching the internet for information relating to your dog’s health? Please select all that apply.

No, I do not feel comfortable talking to my veterinary practice about online searches for information relating to my dog’s health

No, searching online for information relating to my dog’s health has never come up in conversation with my veterinary practice

Yes, I have had conversations about online searches for information relating to my dog’s health with my veterinary practice, prompted by my veterinary practice

Yes, I have had conversations about online searches for information relating to my dog’s health with my veterinary practice, prompted by me

Not applicable, I do not search online for information relating to my dog’s health

Not applicable, I have never taken my dog to a veterinary practice

I am not sure

Other (Please specify)

1. In the **future**, how likely are you to instigate a conversation with professionals at your veterinary practice about searching the internet for information relating to your dog’s health?

1 – extremely unlikely

2 – quite unlikely

3 – neutral

4 – quite likely

5 – extremely likely

1. **LOGIC IF 1 OR 2** Please describe **why** you are **likely** to instigate a conversation with professionals at your veterinary practice about searching the internet for information relating to your dog’s health?

Free Text

**Logic if 3 continue to next section**

**LOGIC IF 4 OR 5** Please describe **why** you are **unlikely** to instigate a conversation with professionals at your veterinary practice about searching the internet for information relating to your dog’s health?

Free Text

1. **General information about you and your dog**

**Participant’s Dog Demographic questions:**

1. How long have you personally owned dog(s) for?

Less than 1 year, 1-3years, 4-7years, 8-11 years, 12-15years, over 16 years

1. How many dogs have you previously owned?

1, 2, 3, 4, 5, 6+

1. How many dogs do you currently own?

1, 2, 3, 4, 5, 6+

1. What breed/s are your current dogs? Please select all that apply

Affenpinscher

Afghan Hound

African Boerboel

Airedale Terrier

Akita

Alaskan Klee Kai

Alaskan Malamute

Alaskan Shepherd

American Bulldog

American Bullnese

American Bully

American Cockapoo

American Cocker Spaniel

American Miniature Shepherd Dog

Australian Cattle Dog

Australian Goldendoodle

Australian Kelpie

Australian Labradoodle

Australian Shepherd Dog

Auvergne Pointer

Bagle

Basset Hound

Beagle

Beaglier

Bearded Collie

Beardoodle

Beauce Shepherd Dog

Bedipoo

Bedlington Terrier

Bedlington Whippet

Belgian Groenendael Shepherd Dog

Belgian Malinois Shepherd Dog

Belgian Tervuren Shepherd Dog

Bernadoodle

Bernese Mountain Dog

Bichon Frise

Biewer Terrier

Black and Tan Coonhound

Bloodhound

Bocker

Borador

Border Collie

Border Terrier

Bordernese

Borderpoo

Borzoi

Bostipoo

Boston Terrier

Bouvier des Flandres

Boxer

Bracco Italiano

Braque du Bourbonnais

Breed not recorded

Briard

Bull Mastiff

Bull Terrier

Bullador

Bulldane

Bulldog

Cairn Terrier

Canadian Inuit Dog

Cardigan Welsh Corgi

Catalan Sheepdog

Cavachi

Cavachichon

Cavachon

Cavador

Cavajack

Cavalier King Charles Spaniel

Cavapom

Cavapoo

Cavapoochon

Cavatzu

Chesapeake Bay Retriever

Chi-Poo

ChiChi

Chichon

Chihuahua

Chinese Crested

Chinese Shar-Pei

Chorkie

Chow Chow

Chuandong Hound

Chug

Clumber Spaniel

Cockador

Cockalier

Cockapoo

Cocker Jack

Cocker Spaniel

Cockeranian

Collie

Coltriever

Companion Spaniel

Corgidor

Coton De Tulear

Cotonese

Crestepoo

Crossbreed

Curly Coated Retriever

Dalmatian

Dandie Dinmont Terrier

Dashalier

Dogue de Bordeaux

Dorkie

Double Doodle

Doxiepoo

English Setter

English Springer Spaniel

English Toy Terrier

Entlebucher Mountain Dog

Eurasier

Fauve de Bretagne Basset

Field Spaniel

Finnish Lapphund

Flat Coated Retriever

Fourche Terrier

French Bulldog

French Korthals Pointing Wire-Haired Griffon

French Water Dog

Frug

Gerberian Shepsky

German Shepherd Dog

German Short-Haired Pointer

German Spitz

German Wire-Haired Pointer

Giant Schnauzer

Goldador

Golden Retriever

Goldendoodle

Gordon Setter

Great Dane

Greatweimar

Greyhound

Griffon Bruxellois

Griffondor

Havachon

Havanese

Highland Maltie

Hungarian Pumi

Hungarian Vizsla

Hungarian Water Dog

Hush Bassett

Huskydor

Irish Doodle

Irish Red and White Setter

Irish Red Setter

Irish Water Spaniel

Irish Wolfhound

Italian Greyhound

Italian Mastiff

Italian Spinone

Jack Russell Terrier

Jack-A-Poo

Jack-Chi

Jack-Tzu

Jackabee

Jackador

Jackarainian

Jackpoochon

Jackshund

Japanese Inu Akita

Japanese Shiba Inu

Japanese Spitz

Jug

Keeshond

King Charles Spaniel

Kooiker Dog

Koolie

Kyi-Leo

La-Chon

Labradoodle

Labrador Retriever

Labralas

Labstaff

Lagotto Romagnolo

Lakedoodle

Lakeland Terrier

Lancashire Heeler

Leonberger

Lhasa Apso

Lhasapoo

Lhatese

Long-Haired Chihuahua

Lowchen

Lucas Terrier

Lurcher

Mal-Shi

Malt Russell

Maltese

Malti-Poo

Maltichon

Maltipom

Manchester Terrier

Maremma Sheepdog

Mastiff

Mastweiller

Miniature Bull Terrier

Miniature Dachshund

Miniature Long-Haired Dachshund

Miniature Pinscher

Miniature Poodle

Miniature Schnauzer

Miniature Smooth-Haired Dachshund

Miniature Wire-Haired Dachshund

Morkie

Munsterlander Large Pointer

NAID Wolfdog

New Zealand Sheepdog

Newfoundland

Newfypoo

Norfolk Terrier

Northern Inuit Dog

Norwegian Elkhound

Norwich Terrier

Nova Scotia Duck Tolling Retriever

Old English Sheepdog

Olde English Bulldogge

Papillon

Parson Russell Terrier

Patterdale Terrier

PatterJack

PatterPoo

Pekalier

Pekingese

Pembroke Welsh Corgi

Petit Basset Griffon Vendeen

Picardy Spaniel

Pointer

Pomapoo

Pomchi

Pomchon

Pomeranian

Pomsky

Poochon

Poogle

Portuguese Pointer

Portuguese Water Dog

Pudelpointer

Pug

Pugalier

Pugapoo

Pughasa

PugTzu

Rhodesian Ridgeback

Rottador

Rottweiler

Saint Bernard

Saluki

Samoyed

Schipperke

Schnauzer

Schnoodle

Schnorky

Scoland Terrier

Scottish Deerhound

Scottish Rough Collie

Scottish Smooth Collie

Scottish Terrier

Sealyham Terrier

Sharpoo

Sheepdog

Shepadoodle

Shepkita

Sheprador

Shetland Sheepdog

ShiChi

Shichon

Shichonpoo

Shih Apso

Shih-Poo

Shih-tzu

Shihpoo spitz

Shiranian

Shollie

Short-Haired Chihuahua

Siberian Husky

Silken Windhound

Slovakian Rough-Haired Pointer

Smooth Fox Terrier

Soft-Coated Wheaten Terrier

Spanish Water Dog

Speagle

Springador

Springer Spaniel - Unspecified

Springerdoodle

Sprocker

Sprockerdor

Sprockerpoo

Sprointer

Sprollie

Stabyhound

Staffordshire Bull Terrier

Standard Doberman Pinscher

Standard Long-Haired Dachshund

Standard Poodle

Standard Smooth-Haired Dachshund

Standard Wire-Haired Dachshund

Sussex Spaniel

Swiss White Shepherd Dog

Tamaskan Husky

Teckel Dachshund

Tibetan Mastiff

Tibetan Spaniel

Tibetan Terrier

Toy German Spitz

Toy Poodle

Utonagan

Weimaraner

Welsh Springer Spaniel

Welsh Terrier

Weshi

West Highland White Terrier

Westiepoo

Whippet

Whoodle

Wire-Haired Fox Terrier

Wire-Haired Hungarian Vizsla

Yoranian

Yorkie Russell

Yorkipoo

Yorkshire Terrier

1. What ages (years) are your current dogs? Select all that apply

1, 2, 3, 4, 5, 6, 7, 8, 9, 10, 11, 12, 13, 14, 15, 16, 17, 18, 19, 20, 21+

**Please answer the following questions about your current dog (or if you have more than one, your dog whose name starts with a letter that is earliest in the alphabet)**

1. Is your current dog registered at a veterinary practice?

Yes

No – I have not registered them as yet, but I intend to in the future

No – I have not registered them and do not intend to unless they become ill

No – I have tried to register them but have been unable to find a practice that will register us

1. How often do you visit your veterinary practice on average for routine health care (e.g. neutering, vaccination, microchipping, routine check-ups, worming treatments, etc.)?

Weekly, monthly, every 3 months, every 6 months, once a year, less than once a year.

1. How often do you visit your veterinary practice on average for your dog’s health problems NOT including any routine or preventative care (e.g., neutering, vaccination, microchipping, routine check-ups, worming treatments, etc.)?

Weekly, monthly, every 3 months, every 6 months, once a year, less than once a year.

1. Is your dog insured?

Yes

Yes – at the moment, but we are undecided whether to continue with it at the next renewal

No – and I do not plan to insure them

No – but I plan to insure them in the future

No – they were insured but I have since cancelled or did not renew their policy

No – they came with 4 weeks free insurance as a puppy, but I did not continue with this

No – I have never heard of pet insurance

No – other (please specify)

1. Is your dog vaccinated?

Yes – just their initial course of vaccinations

Yes – their initial course of vaccinations and an annual booster

No – not yet, but I plan to in the future

No – not yet, I haven’t decided

No – I have chosen not to vaccinate my dog and don’t plan to in the future

1. Do you regularly flea and worm your dog?

Yes – I acquire these through my vet

Yes – I acquire these from a shop, but without prescription

Yes – but I use natural/homeopathic remedies

No – I have chosen not to use anything regularly to prevent fleas and/or worms

1. Has your dog been diagnosed with any long-term health conditions?

YES or NO

1. **LOGIC -IF YES,** Please can you outline them

FREE TEXT

**Participant demographic questions:**

1. How old are you?

18-24, 25-34, 35-44, 45-54, 55-64, 65-74, 75-84, 85+, prefer not to say

1. What gender do you identify as?

Male, Female, Non-Binary, Other, prefer not to say

1. In which region of the UK do you reside?

ABERDEENSHIRE

ANGUS

ANTRIM

ARGULL & BUTE

ARMAGH

AYRSHIRE

BANDDSHIRE

BATH AND NORTH EAST SOMERSET

BEDFORDSHIRE

BERKSHIRE

BERWICKSHIRE

BLAENAU GWENT

BRIDGEND

BRISTOL

BUCKINGHAMSHIRE

CAERPHILLY

CAITHNESS

CAMBRIDGESHIRE

CARDIFF

CARMARTHENSHIRE

CEREDIGION

CHESIRE

CLACKMANNANSHIRE

CONWY

CORDERS

CORNWALL

COUNTY DURHAM

CUMBRIA

DENBIGHSHIRE

DERBYSIDE

DEVON

DOWN

DUMFRIES & GALLOWAY

DUNBARTONSHIRE

EAST AYRSHIRE

EAST DUNBARTONSHIRE

EAST LOTHIAN

EAST RENFREWSHIRE

EAST RIDING OF YORKSHIRE

EAST SUSSEX

ESSEX

FERMANANGH

FIFE

FLINTSHIRE

GLOUCESTERSHIRE

GREATER LONDON

GREATER MANCHESTER

GWYNEDD

HAMPSHIRE

HEREFORDSHIRE

HERTFORDSHIRE

HIGHLAND

INVERCLYDE

ISLE OF ANGLESEY

ISLE OF WIGHT

ISLES OF SCILLY

KENT

KINCARDINESHIRE

LANCASHIRE

LEICESTERSHIRE

LICOLNSHIRE

LONDONDERRY

MERSEYSIDE

MERTHYR TYDFIL

MIDLOTHIAN

MONMOUTHSHIRE

MORAY

NEATH PORT TALBOT

NEWPORT

NORFOLK

NORTH AYRSHIRE

NORTH LANARKSHIRE

NORTH SOMERSET

NORTH YORKSHIRE

NORTHAMPTONSHIRE

NORTHUMBERLAND

NOTTINGHAMSHIRE

ORKNEY

OXFORDSHIRE

PEMBROKESHIRE

PERTH & KINROSS

POWYS

RENFREWHIRE

RHONDDA CYNON TAFF

RUTLAND

SHETLAND

SHROPSHIRE

SOMERSET

SOUTH AYRSHIRE

SOUTH GLOUCESTERSHIRE

SOUTH LANARKSHIRE

SOUTH YORKSHIRE

STAFFORDSHIRE

STIRLINGSHIRE

SUFFOLK

SURREY

SWANSEA

TORFAEN

TYNE & WEAR

TYRONE

VALE OF GLAMORGAN

WARWICKSHIRE

WEST DUNBARTONSHIRE

WEST LOTHIAN

WEST MIDLANDS

WEST SUSSEX

WEST YORKSHIRE

WESTERN ISLES

WILTSHIRE

WORCESTERSHIRE

WREXHAM

OTHER (PLEASE SPECIFY)

1. What is your highest level of education?

GCSEs/O levels, AS Levels, A-levels, diploma in higher education, university higher degree, first degree level qualification (including foundation degrees, graduate membership of a professional institute, PGCE), welsh baccalaureate, international baccalaureate, higher grade/advanced higher (Scotland), certificate of sixth year studies, prefer not to say

1. What is your overall household income?

Up to £10,000; £10,001 - £20,000; £20,001 - £30,000; £30,001 - £40,000; £40,001 - £50,000; £50,001 - £60,000; £60,001 - £70,000; £70,001 - £80,000; £80,001 - £90,000; £90,001 - £100,000; more than £100,001; prefer not to say

1. Are you employed in the canine and/or animal care sector (e.g. veterinary nurse, dog groomer, dog trainer, etc.)

YES OR NO OR I’M NOT SURE

1. If yes, Which canine and/or animal care sector are you employed in?

Veterinary surgeon, Veterinary nurse, Animal care assistant, Veterinary scientist, Dog behaviourist, Dog trainer, Dog day-care/boarding kennels, Dog walker, Dog groomer, Rehoming centre staff, Pet shop worker, Other (please specify)

1. **THANK YOU AND ACCESS TO RESOURCES**

Thank you very much for taking part in our study, your time and effort in completing this survey is very much appreciated. We understand that this study may have worried some owners and so we have collated information sources from major animal welfare organisations below.

**Worried about your dog’s health?**

To find a local veterinary surgeon, please visit the following website:

<https://findavet.rcvs.org.uk/home/>

**Worried about your dog’s behaviour?**

If you are worried about a behavioural problem your dog has developed, talk to your vet about being referred to an accredited animal behaviorist:

<https://fabclinicians.org/find-a-behaviourist/>

**Worried about your own mental health?**

Dog ownership can be an emotional time and unfortunately things can go wrong causing owners heartbreak, guilt and worry. In addition, the COVID-19 lockdown period has been a challenging time for mental health across the UK. If you would like support or advice regarding your own mental health, the following websites may be useful:

- - <https://www.nhs.uk/oneyou/every-mind-matters/>
  - [https://www.samaritans.org](https://www.samaritans.org/)

**S3 Table.** **Clinical vignettes presented to respondents, including condition prevalence, presentation type, dog signalment, and vignette text**

| **Vignette #** | **Condition** | **Condition prevalence**  **(%)** | **Present-ation type** | **Dog signalment** | **Vignette** |
| --- | --- | --- | --- | --- | --- |
| 10A | Mammary Mass | 0.46% | Chronic | ‘Floss’ is a 9 year old, unneutered female Bichon Frise. | Floss has got a 2 cm mass by a nipple on her tummy. It feels hard when you touch it. This is the first time it has been noticed. Floss is otherwise well in herself. It is 4pm on a Thursday. |
| 10B | Corneal Ulcer | 0.77% | Emergency | ‘Dave' is a 6 year old, neutered male French Bulldog | Dave has been blinking more with his left eye for the last few days. Today the eye looks red and he seems painful with the eye. You have noticed that the surface of the eye has a mark on it. It is 3pm on a Thursday. |
| 10C | Aural Haematoma | 0.24% | Chronic | ‘Delilah' is a 11 year old, neutered female Bassett Hound X. | Delilah has had problems with her ears (ear infections) on and off all of her life. Her most recent infection was a week ago, but she is having treatment for it. She has been scratching her right ear more the last few days. Today you have noticed an orange sized lump under the skin on her right ear and it looke very red. It does not seem to be causing her any discomfort. This is the first time a lump like this has appeared on her ear. it is 1pm on a Tuesday. |
| 1A | Kennel Cough | 0.96% | Acute | “Rosie” Is a 5 year old neutered female, West Highland White Terrier | Rosie went to an event over the weekend and there were lots of dogs interacting. She has now developed cough also is gagging, sneezing and retching and now seems more tired than usual. It is midday on Tuesday. |
| 1B | Epilepsy | 0.20% | Acute | "Holly" Is a 1 year old, un-neutered female Border Collie | Holly had an unusual episode whilst at home where she fell to the floor, became unresponsive, and was foaming at mouth. She remained unresponsive and her legs were paddling (twitching/kicking legs) for around 60seconds. She then stood up and appeared to be back to normal within 5 minutes. She had never done this before. It is 9pm on a Tuesday evening. |
| 1C | Cataracts | 0.94% | Chronic | "Buddy" is a 9 year old, neutered male Minature Schnauzer | Buddy is diabetic and has been on medication for six months. The last few days, Buddy has been bumping into things and isn't catching treats as well as he normally does. The eyes look a little cloudy. It is 3pm on a Thursday. |
| 2A | Patella Luxation | 1.04% | Acute | “George” Is a 6month old un-neutered, male Maltipoo (Maltese X Poodle) | George has been on and off skipping/limping on the right hind leg during his walk this morning. This is the first time he has ever done this. It is 9am on a Friday. |
| 2B | Pyometra | 0.26% | Emergency | "Ginny" is a 8 year old, un-neutered female Llhasa Apso | Ginny has been not herself for the last two days. She has vomited 3 times, mostly bile. She has not been eating her food. She has little energy and feels cold to the touch. There has been a small amount of discharge from her vulva. Her last season (heat) was two months ago. It is 11am on a Saturday. |
| 2C | Dry Eye (Keratoconjinctivitis sicca) | 0.33% | Chronic | ‘Purdy' is a 8 year old, un-neutered female British Bulldog. | Purdy has her eyes cleaned daily but she has been rubbing her eyes since yesterday. There has been some thicker sticky discharge from her eyes. She has never had this before. It is 10 am on a Tuesday. |
| 3A | Tail pull/swimmers tail | 0.22% | Acute | "Mouse" is a 7 year old, un-neutered female Lurcher | Mouse was out on a walk this morning and ran off out of sight whilst chasing a scent. When she came back, the end of her tail was limp and she doesn't like it being touched. It is 9am on a Tuesday |
| 3B | Gastrointestinal Foreign Body | 1.27% | Emergency | ‘Alfie' is a 5 year old, neutered male Labradoodle (Labrador X Poodle) | Alfie has been vomiting every hour through the night. He ate his evening meal yesterday but vomited it all up. This morning he again ate his breakfast, but then vomited it up shortly afterwards. He seems a little quieter than usual. Yesterday, he went on a long walk, and he was off the lead for most of it. Previously he has been a scavenger. It is 8am on a Sunday. |
| 3C | Mast Cell Tumour | 0.27% | Chronic | ‘Bodhi' is a 6 year old, neutered male Staffordshire Bull Terrier | Bodhi has a lump on his right side on the skin near his last rib. It fluctuates in nature - sometimes it is a defined lump, and other times it is flattened and small. Sometimes it looks red. Bodhi is fine in himself and is eating/toileting normally. The lump was first noticed a week ago. It is 10am on a Tuesday. |
| 4A | Acute Moist Dermatitis (Hotspot) | 1.46% | Acute | "Honey" is a 6 year old, neutered female Labrador | Honey came to lie next to you on the sofa and you notice a raised red circular ulcerative sore on right side of her face. This is the first time you have noticed this, and she is otherwise well in herself. It is 6pm on a Sunday. |
| 4B | Glaucoma | 0.09% | Emergency | "Mickey" is a 12 year old, neutered male English Springer Spaniel | For the last couple of days, Mickey's left eye has appeared intermittently cloudy with extreme red around the edges. The eye appears watery, and you can't see into the eye. He keeps going to his bed and doesn't want to play this morning. It is 8am on a Saturday. |
| 4C | Round Worms | 0.39% | Chronic | ‘Maisy' is a 8 month old, un-neutered female English springer spaniel. | Maisy is a bright, happy puppy. She is well in herself and always seems hungry. She has just passed faeces and there is spaghetti like strands in it. It is 4pm on a Monday. |
| 5A | Anal Gland Infection | 4.80% | Acute | ‘Summer' is a 7 year old, neutered female Cavapoo (Cavalier spaniel X Poodle) | Summer has been scooting on her bottom the last two days and today has started trying to lick/scratch her bottom. She has her anal glands emptied every couple of months but has never been this bothered by them. There is a foul smell coming from her bottom. it is 11am on a Thursday. |
| 5B | Laceration to Paw Pad | 0.72% | Emergency | "Lexi" is a 3 year old, un-neutered female German Shorthaired Pointer | Lexi has been out on a walk this morning and caught her foot. She was running and went out of sight. She has now come back and is non-weight bearing on her right front leg. There is blood dripping from the right front paw. It is 7.30am on a Tuesday. |
| 5C | Diabetes | 0.34% | Chronic | "Snowy" is a 10year old, neutered female West Highland White Terrier | Snowy has been drinking lots more over the past week and has urinated overnight (this is the first time she has done this). The urine smells almost sweet. She feels as if she may have lost some weight, but her appetite is good, and she is eating well. It is 7am on a Wednesday. |
| 6A | Pancreatitis | 0.35% | Acute | "Monty" is a 5 year old, un-neutered male English Cocker Spaniel. | Monty was sick three times on Friday. He has not been himself since. He is eating, but not as much as normal. He has been in a praying position (stretching out as if uncomfortable) a couple of times today. He also grunted when lifted. It is 10am on a Saturday. |
| 6B | Flea Infestation | 2.05% | Chronic | ‘Nel' is a 6 month old, un-neutered female Hungarian Vizsla | Nel has been scratching a bit more than usual the past two days. There have been some black dirt-like specs on her coat. She is otherwise well in herself. It is 12pm on a Monday. |
| 6C | Osteoarthritis | 2.34% | Chronic | ‘Wilbur' is a 10 year old, neutered male Labrador | Wilbur was limping consistently yesterday on his front legs - this is the first time you have noticed this. Over some time, he has been slow and reluctant when rising from his bed. He seems like he is also stiff when walking at times. It is 10am on a Wednesday. |
| 7A | Otitis Externa | 7.30% | Acute | ‘Bella' is an 8 month old, un-neutered female Beagle. | Bella has been shaking her head more over the last couple of days. She has now started scratching her ears and you've noticed her some dark brown wax from her right ear. She also smells worse than usual. This is the first time you've noticed this. It is 2pm on a Friday. |
| 7B | False Pregnancy (pseudocyesis) | 0.40% | Acute | ‘Willow' is a 1 year old, un-neutered female Whippet. | Willow has been whining/crying on and off overnight. Her nipples are very swollen, and she keeps taking toys to her bed. Her first season was about a month ago. It is 9am on a Wednesday. |
| 7C | Dental Disease | 12.50% | Chronic | ‘Jodie' is a 11 year old, neutered female Yorkshire Terrier | Jodie has been quieter than usual for the last two days. She is eating less than usual, and you can smell a foul odour coming from her mouth. It is 3pm on a Friday. |
| 8A | Urinary Tract Infection | 0.59% | Acute | ‘Kira' is a 3 year old, un-neutered female Shar-pei. | Kira has been urinating more frequently today. She has urinated inside, which is not normal for her, and it is red/blood tinged. She is still eating and drinking but lying in her bed more than usual. It is 7pm on a Tuesday. |
| 8B | Heat Stroke | 0.25% | Emergency | ‘Bruno' is a 5 year old, un-neutered male Pug. | Bruno has always made a lot of noise whilst breathing and snores when he sleeps. It is a really warm day and Bruno is panting lots despite not exercising. He cannot settle and feel very warm to the touch. It is 2pm on a Saturday. |
| 8C | Interdigital Cyst | 0.30% | Acute | ‘Teddie' is a 6 year old, neutered female Jack Russell Terrier. | Teddie has been really licking and chewing her back left paw for the past two days. The paw is now sore, and you can feel a lump in between two of the toes on that paw. It is 7am on a Thursday in Winter. |
| 9A | Atopy | 1.15% | Acute | ‘Obi' is a 1 year old, neutered male West Highland White Terrier. | Obi has been itching and scratching lots over the last few days. He has itched some areas so much they are red and sore. He seems very subdued in comparison to normal. He is up to date with flea and worm treatment. It is 12pm on a Wednesday. |
| 9B | Congestive Heart Failure | 0.39% | Emergency | ‘Felicity' is a 7 year old, neutered female Cavalier King Charles Spaniel | Felicity has been slowly getting more reluctant to exercise and today she refuses to exercise. She has a newly developed cough and her abdomen feel more swollen than usual. It is 5pm on a Monday. |
| 9C | Torn Nail | 1.38% | Acute | ‘Milo' is a 1 year old, un-neutered male Boxer. | Milo was playing in the garden and now he has come back inside he is lame on his left front leg. He won't let anyone touch it and there is some blood around the dew claw. He is whimpering and seems very painful. It is 5pm on a Friday. |

**S3 Table: Example accuracy scoring matrix used to assess UK dog owners condition identification accuracy for Kennel Cough.**

| **Unique participant free-text answer** | **Accuracy** | **Certainty** | **Score** |
| --- | --- | --- | --- |
| Cold | 1 | 3 | 3 |
| Kennel cough | 3 | 3 | 9 |
| Upper respiratory tract infection | 2 | 3 | 6 |
| A virus | 1 | 3 | 3 |
| Respiratory infection | 2 | 3 | 6 |
| Something stuck in throat or airway irritation | 0 | 2 | 0 |
| I would be wondering if it might be kennel cough | 3 | 2 | 6 |
| I have no idea. Poss ingested foreign body/ allergic reaction? | 0 | 1 | 0 |
| Canine flu | 1 | 3 | 3 |
| Something possibly stuck | 0 | 2 | 0 |
| Possibly kennel cough | 3 | 2 | 6 |
| Potentially kennel cough | 3 | 2 | 6 |
| Kennel caugh | 3 | 3 | 9 |
| Pi | 2 | 3 | 6 |
| Kennel cough. Isolate from other dogs. There's no point going to the vet unless it gets worse as they can only treat the secondaries. Antibiotics are NOT the answer. | 3 | 3 | 9 |
| Cv19 | 1 | 3 | 3 |
| Whooping cough | 1 | 3 | 3 |
| Virus or potentially kennel cough | 2 | 2 | 4 |
| Flu | 1 | 3 | 3 |
| Kennal cough | 3 | 3 | 9 |

**S4 Table: Responses (n) to each clinical vignette**

| **Vignette number** | **Condition** | **Number of responses (n)** |
| --- | --- | --- |
| 10A | Mammary Mass | 149 |
| 10B | Corneal Ulcer | 149 |
| 10C | Aural Haematoma | 149 |
| 1A | Kennel Cough | 137 |
| 1B | Epilepsy | 137 |
| 1C | Cataracts | 137 |
| 2A | Patella Luxation | 158 |
| 2B | Pyometra | 158 |
| 2C | Dry Eye (Keratoconjinctivitis sicca) | 158 |
| 3A | Tail pull/swimmers tail | 241 |
| 3B | Gastrointestinal Foreign Body | 241 |
| 3C | Mast Cell Tumour | 241 |
| 4A | Acute Moist Dermatitis (Hotspot) | 169 |
| 4B | Glaucoma | 169 |
| 4C | Round Worms | 169 |
| 5A | Anal Gland Infection | 175 |
| 5B | Laceration to Paw Pad | 175 |
| 5C | Diabetes | 175 |
| 6A | Pancreatitis | 167 |
| 6B | Flea Infestation | 167 |
| 6C | Osteoarthritis | 167 |
| 7A | Otitis Externa | 296 |
| 7B | False Pregnancy (pseudocyesis) | 296 |
| 7C | Dental Disease | 296 |
| 8A | Urinary Tract Infection | 182 |
| 8B | Heat Stroke | 182 |
| 8C | Interdigital Cyst | 182 |
| 9A | Atopy | 179 |
| 9B | Congestive Heart Failure | 179 |
| 9C | Torn Nail | 179 |

**S5 Table: Univariable analysis (Chi^2^) for UK first-time dog owners and top three most frequently used canine health information-sources for health information for their dog.**

| **Top 3 most frequently used canine health information-sources N=1758** | **Sub category** | **Not a 1^st^ time owner**  **N (%)** | **1^st^ time dog owner**  **N (%)** | **X^2^** | **P value** |
| --- | --- | --- | --- | --- | --- |
| **Existing knowledge/experience** | **No** | **378 (24.3)** | **82 (40.8)** | **25.143** | **<0.001** |
|  | **Yes** | **1179 (75.7)** | **119 (59.2)** |  |  |
| Directly contact my local vets/veterinary professionals in practice | No | 598 (38.4) | 86 (42.8) | 1.436 | 0.231 |
|  | Yes | 959 (61.6) | 115 (57.2) |  |  |
| **Internet search** | **No** | **825 (53.0)** | **70 (34.8)** | **23.493** | **<0.001** |
|  | **Yes** | **732 (47.0)** | **131 (65.2)** |  |  |
| Books | No | 1486 (95.4) | 197 (98.0) | 2.879 | 0.090 |
|  | Yes | 71 (4.6) | 4 (2.0) |  |  |
| **Free vet/nurse hotlines** | **No** | **1392 (89.4)** | **169 (84.1)** | **5.069** | **0.024** |
|  | **Yes** | **165 (10.6)** | **32 (15.9)** |  |  |
| Directly contact animal care staff (e.g. Groomer, dog walker, kennel assistant) | No | 1513 (97.2) | 191 (95.0) | 2.762 | 0.097 |
|  | Yes | 44 (2.8) | 10 (5.0) |  |  |
| **Directly contact pet shop staff** | **No** | **1553 (99.7)** | **198 (98.5)** | **6.853** | **0.009** |
|  | **Yes** | **4 (0.3)** | **3 (1.5)** |  |  |
| Directly contact veterinary physiotherapist | No | 1487 (95.5) | 190 (94.5) | 0.386 | 0.534 |
|  | Yes | 70 (4.5) | 11 (5.5) |  |  |
| Directly contact breeder | No | 1450 (93.1) | 187 (93.0) | 0.002 | 0.961 |
|  | Yes | 107 (6.9) | 14 (7.0) |  |  |
| Podcasts | No | 1555 (99.9) | 201 (100.0) | 0.258 | 0.611 |
|  | Yes | 2 (0.1) | 0 (0.0) |  |  |
| Magazines | No | 1557 (100.0) | 200 (99.5) | 7.751 | 0.05 |
|  | Yes | 0 (0.0) | 1 (0.5) |  |  |
| TV Shows | No | 1553 (99.7) | 200 (99.5) | 0.363 | 0.547 |
|  | Yes | 4 (0.3) | 1 (0.5) |  |  |
| Radio | No | 1556 (99.9) | 201 (100.0) | 0.129 | 0.719 |
|  | Yes | 1 (0.1) | 0 (0.0) |  |  |
| **Friends or family members (who are not veterinary professionals)** | **No** | **1328 (85.3)** | **160 (79.6)** | **4.434** | **0.035** |
|  | **Yes** | **229 (14.7)** | **41 (20.4)** |  |  |
| Friends or family members (who are veterinary professionals) | No | 1211 (77.8) | 163 (81.1) | 1.147 | 0.284 |
|  | Yes | 346 (22.2) | 38 (18.9) |  |  |
| **Online group(s) (related to dog breed/crossbreed)** | **No** | **1326 (85.2)** | **154 (76.6)** | **9.768** | **0.002** |
|  | **Yes** | **231 (14.8)** | **47 (23.4)** |  |  |
| Online group(s) (related to dog health condition, if applicable) | No | 1329 (85.4) | 175 (87.1) | 0.420 | 0.517 |
|  | Yes | 228 (14.6) | 26 (12.9) |  |  |
| Online group(s) (general dog related groups) | No | 1433 (92.0) | 179 (89.1) | 2.078 | 0.149 |
|  | Yes | 124 (8.0) | 22 (10.9) |  |  |
| Online group(s)(not related to dogs) | No | 1553 (99.7) | 201 (100.0) | 0.518 | 0.472 |
|  | Yes | 4 (0.3) | 0 (0.0) |  |  |
| Scientific publications | No | 1552 (99.7) | 201 (100.0) | 0.647 | 0.421 |
|  | Yes | 5 (0.3) | 0 (0.0) |  |  |
| **Telemedicine** | **No** | **1557 (100.0)** | **200 (99.5)** | **7.751** | **0.005** |
|  | **Yes** | **0 (0.0)** | **1 (0.5)** |  |  |
| Webinars | No | 1556 (99.9) | 201 (100.0) | 0.129 | 0.719 |
|  | Yes | 1 (0.1) | 0 (0.0) |  |  |

**S6 Table: Univariable analysis (Chi^2^) for UK chronically-ill dog owners and top three most frequently used canine health information-sources for health information for their dog.**

| **Top 3 most frequently used canine health information-sources n=1757** | **Sub category** | **Doesn’t own dog with long-term condition n (%)** | **Own dog with long-term condition n (%)** | ***X^2^*** | **P value** |
| --- | --- | --- | --- | --- | --- |
| Existing knowledge/experience | No | 327 (26.0) | 132 (26.6) | 0.068 | 0.794 |
|  | YES | 933 (74.0) | 365 (73.4) |  |  |
| Directly contact my local vets/veterinary professionals in practice | No | 502 (39.8) | 183 (36.8) | 1.367 | 0.242 |
|  | Yes | 758 (60.2) | 314 (63.2) |  |  |
| Internet search | No | 651 (51.7) | 243 (48.9) | 1.097 | 0.295 |
|  | Yes | 609 (48.3) | 254 (51.1) |  |  |
| Books | No | 1206 (95.7) | 475 (95.6) | 0.017 | 0.896 |
|  | Yes | 54 (4.3) | 22 (4.4) |  |  |
| Free vet/nurse hotlines | No | 1115 (88.5) | 446 (89.7) | 0.559 | 0.455 |
|  | Yes | 145 (11.5) | 51 (10.3) |  |  |
| Directly contact animal care staff (e.g. groomer, dog walker, kennel assistant) | No | 1215 (96.4) | 487 (98.0) | 2.858 | 0.091 |
|  | Yes | 45 (3.6) | 10 (2.0) |  |  |
| Directly contact pet shop staff | No | 1254 (99.5) | 496 (99.8) | 0.679 | 0.410 |
|  | Yes | 6 (0.5) | 1 (0.2) |  |  |
| Directly contact veterinary physiotherapist | No | 1206 (95.7) | 470 (94.6) | 1.066 | 0.302 |
|  | Yes | 54 (4.3) | 27 (5.4) |  |  |
| **Directly contact breeder** | **No** | **1159 (92.0)** | **476 (95.8)** | **7.925** | **0.005** |
|  | **Yes** | **101 (8.0)** | **21 (4.2)** |  |  |
| **Podcasts** | **No** | **1260 (100.0)** | **495 (99.6)** | **5.076** | **0.024** |
|  | **Yes** | **0 (0.0)** | **2 (0.4)** |  |  |
| Magazines | No | 1260 (100.0) | 496 (99.8) | 2.537 | 0.111 |
|  | Yes | 0 (0.0) | 1 (0.2) |  |  |
| TV Shows | No | 1255 (99.6) | 497 (100.0) | 1.978 | 0.160 |
|  | Yes | 5 (0.4) | 0 (0.0) |  |  |
| Radio | No | 1259 (99.9) | 497 (100.0) | 0.395 | 0.530 |
|  | Yes | 1 (0.1) | 0 (0.0) |  |  |
| **Friends or family members (who are not veterinary professionals)** | **No** | **1052 (83.5)** | **435 (87.5)** | **4.458** | **0.035** |
|  | **Yes** | **208 (16.5)** | **62 (12.5)** |  |  |
| Friends or family members (who are veterinary professionals) | No | 978 (77.6) | 396 (79.7) | 0.886 | 0.346 |
|  | Yes | 282 (22.4) | 101 (20.3) |  |  |
| Online group(s) (related to dog breed/crossbreed) | No | 1055 (83.7) | 423 (85.1) | 0.509 | 0.476 |
|  | Yes | 205 (16.3) | 74 (14.9) |  |  |
| **Online group(s) (related to dog health condition, if applicable)** | **No** | **1112 (88.3)** | **392 (78.9)** | **25.445** | **<0.001** |
|  | **Yes** | **148 (11.7)** | **105 (21.1)** |  |  |
| **Online group(s) (general dog related groups)** | **No** | **1145 (90.9)** | **466 (93.8)** | **3.906** | **0.048** |
|  | **Yes** | **115 (9.1)** | **31 (6.2)** |  |  |
| Online group(s)(not related to dogs) | No | 1257 (99.8) | 496 (99.8) | 0.021 | 0.884 |
|  | Yes | 3 (0.2) | 1 (0.2) |  |  |
| Scientific publications | No | 1256 (99.7) | 496 (99.8) | 0.170 | 0.680 |
|  | Yes | 4 (0.3) | 1 (0.2) |  |  |
| Telemedicine | No | 1259 (99.9) | 497 (100.0) | 0.395 | 0.530 |
|  | Yes | 1 (0.1) | 0 (0.0) |  |  |
| Webinars | No | 1260 (100.0) | 496 (99.8) | 2.537 | 0.111 |
|  | Yes | 0 (0.0) | 1 (0.2) |  |  |

**S7 Table: Univariable analysis (Chi^2^) for UK brachycephalic purebred or brachycephalic crossbreed dog owners and top three most frequently used canine health information-sources for health information for their dog.**

| **Top 3 most frequently used canine health information-sources N=1590** | **Sub category** | **Not a brachycephalic breed or crossbreed**  **N (%)** | **Brachycephalic breed or crossbreed**  **N (%)** | ***X^2^*** | **P value** |
| --- | --- | --- | --- | --- | --- |
| Existing knowledge/experience | No | 389 (26.5) | 32 (26.2) | 0.004 | 0.948 |
|  | Yes | 1079 (73.5) | 90 (73.8) |  |  |
| Directly contact my local vets/veterinary professionals in practice | No | 576 (39.2) | 50 (41.0) | 0.144 | 0.704 |
|  | Yes | 892 (60.8) | 72 (59.0) |  |  |
| Internet search | No | 769 (52.4) | 54 (44.3) | 2.976 | 0.085 |
|  | Yes | 699 (47.6) | 68 (55.7) |  |  |
| Books | No | 1403 (95.6) | 114 (93.4) | 1.166 | 0.280 |
|  | Yes | 65 (4.4) | 8 (6.6) |  |  |
| Free vet/nurse hotlines | No | 1301 (88.6) | 108 (88.50 | 0.001 | 0.974 |
|  | Yes | 167 (11.4) | 14 (11.5) |  |  |
| Directly contact animal care staff (e.g. Groomer, dog walker, kennel assistant) | No | 1422 (96.9) | 119 (97.5) | 0.172 | 0.679 |
|  | Yes | 46 (3.1) | 3 (2.5) |  |  |
| Directly contact pet shop staff | No | 1464 (99.7) | 122 (100.0) | 0.333 | 0.564 |
|  | Yes | 4 (0.3) | 0 (0.0) |  |  |
| Directly contact veterinary physiotherapist | No | 1403 (95.6) | 119 (97.5) | 1.066 | 0.302 |
|  | Yes | 65 (4.4) | 3 (2.5) |  |  |
| Directly contact breeder | No | 1352 (92.1) | 118 (96.7) | 3.450 | 0.063 |
|  | Yes | 116 (7.9) | 4 (3.3) |  |  |
| Podcasts | No | 1466 (99.9) | 122 (100.0) | 0.166 | 0.683 |
|  | Yes | 2 (0.1) | 0 (0.0) |  |  |
| Magazines | No | 1467 (99.9) | 122 (100.0) | 0.083 | 0.773 |
|  | Yes | 1 (0.1) | 0 (0.0) |  |  |
| TV Shows | No | 1464 (99.7) | 121 (99.2) | 1.076 | 0.300 |
|  | Yes | 3 (0.3) | 1 (0.8) |  |  |
| Radio | No | 1468 (100.0) | 122 (100.0) | NA | NA |
|  | Yes | 0 (0.0) | 0 (0.0) |  |  |
| Friends or family members (who are not veterinary professionals) | No | 1240 (84.5) | 108 (88.5) | 1.436 | 0.231 |
|  | Yes | 228 (15.5) | 14 (11.5) |  |  |
| Friends or family members (who are veterinary professionals) | No | 1148 (78.2) | 97 (79.5) | 0.113 | 0.737 |
|  | Yes | 320(21.8) | 25 (20.5) |  |  |
| Online group(s) (related to dog breed/crossbreed) | No | 1233 (84.0) | 95 (77.9) | 3.068 | 0.080 |
|  | Yes | 235 (16.0) | 27 (22.1) |  |  |
| Online group(s) (related to dog health condition, if applicable) | No | 1264 (86.1) | 101 (82.8) | 1.020 | 0.313 |
|  | Yes | 204 (13.9) | 21 (17.2) |  |  |
| Online group(s) (general dog related groups) | No | 1354 (92.2) | 108 (88.5) | 2.094 | 0.148 |
|  | Yes | 114 (7.8) | 14 (11.5) |  |  |
| Online group(s) (not related to dogs) | No | 1465 (99.8) | 122 (100.0) | 0.250 | 0.617 |
|  | Yes | 3 (0.2) | 0 (0.0) |  |  |
| Scientific publications | No | 1463 (99.7) | 122 (100.0) | 0.417 | 0.519 |
|  | Yes | 5 (0.3) | 0 (0.0) |  |  |
| Telemedicine | No | 1467 (99.9) | 122 (100.0) | 0.083 | 0.773 |
|  | Yes | 1 (0.1) | 0 (0.0) |  |  |
| Webinars | No | 1468 (100.0) | 122 (100.0) | NA | NA |
|  | Yes | 0 (0.0) | 0 (0.0) |  |  |

**S8 Table:** **Univariable analysis for predictors of UK dog owners’ accuracy of identification of common canine conditions using Mann-Whitney U test.**

| **Variable** | **Sub category** | **N** | **Median accuracy score** | **25-75 percentile** | **Test statistic** | **P value** |
| --- | --- | --- | --- | --- | --- | --- |
| Owns a dog diagnosed with a long term condition | Yes | 1497 | 3 | 0-6 | 2837117 | 0.943 |
|  | No | 3795 | 3 | 0-6 |  |  |
| 1^st^ time owner | Yes | 606 | 3 | 0-6 | 1393909 | 0.427 |
|  | No | 4689 | 3 | 0-6 |  |  |
| Owns a brachycephalic breed or brachycephalic crossbreed | Yes | 366 | 3 | 0-6 | 805082 | 0.864 |
|  | no | 4422 |  |  |  |  |
| **Employed in the canine industry** | **Yes** | **1095** | **4** | **0-6** | **2625029** | **0.001** |
|  | **no** | **4188** | **3** | **0-6** |  |  |
| Used the internet to aid vignette answers | Yes | 957 | 3 | 0-6 | 2009200 | 0.238 |
|  | No | 4299 | 3 |  |  |  |
| **Used their own knowledge to aid vignette answers** | **Yes** | **4593** | **3** | **0-6** | **1657650** | **<0.001** |
|  | **No** | **663** | **2** |  |  |  |
| **Habitually uses their own knowledge** | Yes | 3900 | 3 | 0-6 | 2794067 | 0.115 |
|  | No | 1395 | 3 |  |  |  |
| Habitually contacts their local veterinary practice | Yes | 3234 | 3 | 0-6 | 3331391 | 0.981 |
|  | No | 2061 | 3 |  |  |  |
| **Habitually uses internet searches** | Yes | 2607 | 3 | 0-6 | 3512199 | 0.874 |
|  | No | 2688 | 3 |  |  |  |
| Has dog insurance | Yes | 3447 | 3 | 0-6 | 3255414 | 0.165 |
|  | No | 1848 | 3 |  |  |  |
| Vet Registration | Yes | 5265 | 1 | 0-6 | 96966 | 0.802 |
| Books for vignette answers | No | 5181 | 3 | 0-6 | 191888 | 0.847 |
|  | Yes | 75 |  |  |  |  |
| **Free hotline for vignette answers** | **No** | **5051** | **3** | **0-6** | **459166** | **0.004** |
|  | **Yes** | **205** | **2** |  |  |  |
| Animal Care staff for vignette answers | No | 5205 | 3 | 0-6 | 134444 | 0.868 |
|  | Yes | 51 |  |  |  |  |
| **Pet Shop Staff for vignette answers** | **No** | **5251** | **3** | **0-6** | **20720.5** | **0.019** |
|  | **Yes** | **5** | **6** | **06-Sep** |  |  |
| Physiotherapist for vignette answers | No | 5193 | 3 | 0-6 | 151814.5 | 0.304 |
|  | Yes | 63 | 2 |  |  |  |
| Breeder for vignette answers | No | 5188 | 3 | 0-6 | 177380 | 0.934 |
|  | Yes | 68 |  |  |  |  |
| Podcasts for vignette answers | No | 5252 | 3 | 0-6 | 10654.5 | 0.959 |
|  | Yes | 4 |  | 0-8.25 |  |  |
| Magazines for vignette answers | No | 5251 | 3 | 0-6 | 8043.5 | 0.117 |
|  | Yes | 5 | 0 | 0-3 |  |  |
| TV for vignette answers | No | 5241 | 3 | 0-6 | 37798.5 | 0.788 |
|  | Yes | 15 | 2 |  |  |  |
| **Family and Friends that are not veterinary professionals for vignette answers** | **No** | **5067** | **3** | **0-6** | **440042** | **0.048** |
|  | **Yes** | **189** | **2** |  |  |  |
| Family and Friends that are veterinary professionals for vignette answers | No | 5024 | 3 | 0-6 | 571692.5 | 0.608 |
|  | Yes | 232 |  |  |  |  |
| Breed specific groups for vignette answers | No | 5003 | 3 | 0-6 | 611013 | 0.331 |
|  | Yes | 253 |  |  |  |  |
| Dog health groups for vignette answers | No | 5044 | 3 | 0-6 | 527394 | 0.725 |
|  | Yes | 212 |  |  |  |  |
| **General Dog groups for vignette answers** | **No** | **5102** | **3** | **0-6** | **339562** | **0.003** |
|  | **Yes** | **154** | **0** |  |  |  |
| General Group for vignette answers | No | 5249 | 3 | 0-6 | 17094 | 0.739 |
|  | Yes | 7 |  |  |  |  |
| Advertising for vignette answers | No | 5255 |  |  | 4768 | 0.14 |
|  | Yes | 1 |  |  |  |  |
| Would contact vets despite being not to for the vignettes | No | 5194 | 3 | 0-6 | 143699 | 0.127 |
|  | Yes | 62 | 2 |  |  |  |
| Contacted vets despite being told not to for vignette answers | No | 5250 | 3 | 0-6 | 17035 | 0.717 |
|  | Yes | 6 |  | 0-9 |  |  |
| Miscellaneous for vignette answers | No | 5237 | 3 | 0-6 | 41735.5 | 0.204 |
|  | Yes | 19 | 0 |  |  |  |
| Webinars for vignette answers | No | 5254 | 3 | 0-6 | 6409.5 | 0.573 |
|  | Yes | 2 | 4.5 | 3- |  |  |
| Habitually use books | No | 5067 | 3 | 0-6 | 607164.5 | 0.171 |
|  | Yes | 228 |  | 0-8.25 |  |  |
| Habitually use free hotlines | No | 4704 | 3 | 0-6 | 1364829 | 0.451 |
|  | Yes | 591 |  |  |  |  |
| Habitually contact animal care staff | No | 5130 | 3 | 0-6 | 398345 | 0.178 |
|  | Yes | 165 | 2 |  |  |  |
| Habitually contact pet shop staff | No | 5274 | 3 | 0-6 | 47694 | 0.25 |
|  | Yes | 21 | 2 |  |  |  |
| Habitually contact physiotherapist | No | 5052 | 3 | 0-6 | 618705.5 | 0.826 |
|  | Yes | 243 | 3 |  |  |  |
| Habitually contact breeder | No | 4929 | 3 | 0-6 | 914610.5 | 0.64 |
|  | Yes | 366 |  |  |  |  |
| Habitually use podcasts | No | 5289 | 3 | 0-6 | 20147.5 | 0.231 |
|  | Yes | 6 | 6 | 2.75-6 |  |  |
| Habitually use Magazines | No | 5289 | 3 | 0-6 | 14598 | 0.723 |
|  | Yes | 6 |  |  |  |  |
| Habitually use TV | No | 5277 | 3 | 0-6 | 44663.5 | 0.647 |
|  | Yes | 18 | 1 | 0-6.75 |  |  |
| Habitually use Radio | No | 5292 | 3 | 0-6 | 10607.5 | 0.291 |
|  | Yes | 3 | 6 | 4- |  |  |
| Habitually contact friends and family that are not veterinary professionals | No | 4485 | 3 | 0-6 | 1820110 | 0.923 |
|  | Yes | 810 |  |  |  |  |
| **Habitually contact friends and family that are veterinary professionals** | **No** | **4150** | **3** | **0-6** | **2536591** | **<0.001** |
|  | **Yes** | **1155** |  |  |  |  |
| Habitually use Breed groups | No | 4452 | 3 | 0-6 | 1819313 | 0.141 |
|  | Yes | 843 |  |  |  |  |
| Habitually use Dog health group | No | 4530 | 3 | 0-6 | 1723072 | 0.796 |
|  | Yes | 765 |  |  |  |  |
| **Habitually use General dog group** | **No** | **4854** | **3** | **0-6** | **1005870** | **0.028** |
|  | **Yes** | **441** |  |  |  |  |
| **Habitually use General groups** | **No** | **5283** | **3** | **0-6** | **42566.5** | **0.031** |
|  | **Yes** | **12** | **6** | **2.25-9** |  |  |
| Habitually use Scientific publications | No | 5280 | 3 | 0-6 | 42117 | 0.656 |
|  | Yes | 15 |  | 0-9 |  |  |
| Habitually use Telemedicines | No | 5292 | 3 | 0-6 | 6880 | 0.676 |
|  | Yes | 3 |  | 0- |  |  |
| Habitually use webinars | No | 5292 | 3 | 0-6 | 11293 | 0.184 |
|  | Yes | 3 | 6 | 3- |  |  |

**Table S9:** **Univariable analysis for predictors of UK dog owners’ accuracy of identification of common canine conditions using Kruskall-Wallis H test.**

| **Variable** | **Subcategory** | **N** | **Median accuracy score** | **25-75 percentile** | **Test statistic** | **P value** |
| --- | --- | --- | --- | --- | --- | --- |
| **Owner age** | **18-24 yr** | **153** | **3** | **0-7.5** | **14.872** | **0.038** |
|  | **25-34** | **624** | **3** | **0-6** |  |  |
|  | **35-44** | **804** | **3** | **0-6** |  |  |
|  | **45-54** | **1209** | **2** | **0-6** |  |  |
|  | **55-64** | **1449** | **3** | **0-6** |  |  |
|  | **65-74** | **843** | **3** | **0-6** |  |  |
|  | **75-84** | **150** | **0** | **0-6** |  |  |
|  | **Prefer not to say** | **33** | **4** | **0-7.5** |  |  |
| Gender | Female | 4971 | 3 | 0-6 | 3.784 | 0.286 |
|  | Male | 240 | 2.5 | 0-6 |  |  |
|  | Other | 9 | 3 | 0-9 |  |  |
|  | Prefer not to say | 96 | 2 | 0-6 |  |  |
| **Income** | **Up to £10,000** | **147** | **4** | **0-9** | **17.66** | **0.090** |
|  | **£10,001 - £20,000** | **426** | **3** | **0-6** |  |  |
|  | **£20,001 - £30,000** | **684** | **3** | **0-6** |  |  |
|  | **£30,001 - £40,000** | **534** | **3** | **0-6** |  |  |
|  | **£40,001 - £50,000** | **501** | **3** | **0-6** |  |  |
|  | **£50,001 - £60,000** | **456** | **3** | **0-6** |  |  |
|  | **£60,001 - £70,000** | **324** | **2** | **0-6** |  |  |
|  | **£70,001 - £80,000** | **234** | **3** | **0-6** |  |  |
|  | **£80,001 - £90,000** | **183** | **3** | **0-6** |  |  |
|  | **£90,001 - £100,000** | **207** | **3** | **0-6** |  |  |
|  | **More than £100,000** | **360** | **2** | **0-6** |  |  |
|  | **Prefer not to say** | **1206** | **3** | **0-6** |  |  |
| Education | Level ½ | 963 | 2 | 0-6 | 7.843 | 0.449 |
|  | Level 3 | 984 | 3 | 0-6 |  |  |
|  | Level 4 | 18 | 2.5 | 0-6 |  |  |
|  | Level 5 | 33 | 4 | 0-6 |  |  |
|  | Level 6 | 1482 | 3 | 0-6 |  |  |
|  | Level 7 | 1212 | 2 | 0-6 |  |  |
|  | Level 8 | 138 | 3 | 0-6 |  |  |
|  | Miscellaneous | 162 | 3 | 0-6 |  |  |
|  | Prefer not to say | 279 | 3 | 0-6 |  |  |
| Vaccination | No | 348 | 3 | 0-6 | 2.702 | 0.259 |
|  | Yes | 3258 |  |  |  |  |
|  | Yes- but not every year | 1683 |  |  |  |  |
| Dog Ownership | Less than 1 year | 108 | 1 | 0-6 | 2.665 | 0.752 |
|  | 1-3 years | 408 | 3 |  |  |  |
|  | 4-7 years | 423 | 3 |  |  |  |
|  | 8-11 years | 435 | 3 |  |  |  |
|  | 12-15 years | 477 | 3 |  |  |  |
|  | Over 16 years | 3441 | 3 |  |  |  |
| Routine Vet Visits | Less frequently than once a year | 1098 | 3 | 0-6 | 1.684 | 0.431 |
|  | Miscellaneous | 36 | 4 |  |  |  |
|  | One a year or more frequently | 4158 | 3 |  |  |  |

**S10 Table: Univariable analysis for predictors of UK dog owners’ condition urgency for veterinary care assessments using Chi^2^ test.**

| **Variable**  **New information sources** | **Sub-category** | **Equal to or more urgent** | **Less urgent** | ***X^2^*** | **P value** |
| --- | --- | --- | --- | --- | --- |
| **New source own knowledge** | **No** | **520 (9.9)** | **142 (2.7)** | **17.984** | **<0.00** |
|  | **Yes** | **3232 (61.7)** | **1346 (25.7)** |  |  |
| **New source books** | **No** | **3689 (70.4)** | **1476 928.2)** | **5.751** | **0.016** |
|  | **Yes** | **63 (1.2)** | **12 (0.2)** |  |  |
| **New source Free hotline** | **No** | **3584 (68.4)** | **1451 (27.7)** | **11.236** | **<0.001** |
|  | Yes | 168 (3.2) | 37 (0.7) |  |  |
| New source animal care staff | No | 3713 (70.9) | 1476 (28.2) | 0.600 | 0.439 |
|  | Yes | 39 (0.7) | 12 (0.2) |  |  |
| Pet Shop Staff | No | 3748 (71.5) | 1487 (28.4) | 0.174 | 0.677 |
|  | Yes | 4 (0.1) | 1 (0.0) |  |  |
| **Physiotherapist** | **No** | **3699 (70.6)** | **1478 (28.2)** | **4.919** | **0.027** |
|  | **Yes** | **53 (1.0)** | **10 (0.2)** |  |  |
| Breeder | No | 3703 (70.7) | 1470 (28.1) | 0.078 | 0.780 |
|  | Yes | 49 (0.9) | 18 (0.3) |  |  |
| Podcasts | No | 3748 (71.5) | 1488 (28.4) | 1.588 | 0.208 |
|  | Yes | 4 (0.1) | 0 (0.0) |  |  |
| Magazines | No | 3747 (71.5) | 1488 (28.4) | 1.985 | 0.159 |
|  | Yes | 5 (0.1) | 0 (0.0) |  |  |
| TV | No | 3740 (71.4) | 1485 (28.3) | 0.522 | 0.470 |
|  | Yes | 12 (0.2) | 3 (0.1) |  |  |
| Family and Friends not vets | No | 3620 (69.1) | 1432 (27.3) | 0.185 | 0.667 |
|  | Yes | 132 (2.5) | 56 (1.1) |  |  |
| Family and Friends Vet | No | 3581 (68.3) | 1427 (27.2) | 0.528 | 0.467 |
|  | Yes | 171 (3.3) | 61 (1.2) |  |  |
| Breed specific groups | No | 3566 (68.1) | 1421 (27.1) | 0.479 | 0.489 |
|  | Yes | 186 (3.5) | 67 (1.3) |  |  |
| **Dog health groups** | **No** | **3580 (68.3)** | **1449 (27.7)** | **10.626** | **0.001** |
|  | **Yes** | **172 (3.3)** | **39 (0.7)** |  |  |
| General Dog groups | No | 3632 (69.3) | 1454 (27.7) | 3.116 | 0.078 |
|  | Yes | 120 (2.3) | 34 (0.6) |  |  |
| General Group | No | 3747 (71.5) | 1486 (28.4) | 0.000 | 0.992 |
|  | Yes | 5 (0.1) | 2 (0.0) |  |  |
| Advertising | No | 3751 (71.6) | 1488 (28.4) | 0.397 | 0.529 |
|  | Yes | 1 (0.0) | 0 (0.0) |  |  |
| **Would contact vets despite being not to for the vignettes** | **No** | **3696 (70.5)** | **1482 (28.3)** | **10.813** | **0.001** |
|  | **Yes** | **56 (1.1)** | **6 (0.1)** |  |  |
| Contacted vets despite being told not to | No | 3746 (71.5) | 1488 (28.4) | 2.382 | 0.123 |
|  | Yes | 6 (0.1) | 0 (0.0) |  |  |
| **Miscellaneous** | **No** | **3742 (71.4)** | **1473 (28.2)** | **3.375** | **0.066** |
|  | **Yes** | **10 (0.2)** | **9 (0.2)** |  |  |
| Webinars | No | 3750 (71.6) | 1488 (28.4) | 0.793 | 0.373 |
|  | Yes | 2 (0.0) | 0 (0.0) |  |  |
| **Internet** | **No** | **3031 (57.8)** | **1253 (23.9)** | **8.372** | **0.004** |
|  | **Yes** | **721 (13.8)** | **235 (4.5)** |  |  |

| **Own knowledge** | No | **1020 (19.4)** | **358 (6.8)** | **5.538** | **0.019** |
| --- | --- | --- | --- | --- | --- |
|  | Yes | **2735 (52.1)** | **1134 (21.6)** |  |  |
| Internet | No | 1920 (36.6) | 743 (14.2) | 0.759 | 0.384 |
|  | Yes | 1835 (35.0) | 749 (14.3) |  |  |
| **Vets** | No | **1360 (25.9)** | **685 (13.1)** | **42.180** | **<0.001** |
|  | Yes | 239 (45.6) | 807 (15.4) |  |  |
| Books | No | 3586 (68.3) | 1435 (27.3) | 1.199 | 0.274 |
|  | Yes | 169 (3.2) | 57 (1.1) |  |  |
| Free hotlines | No | 3319 (63.3) | 1343 (25.6) | 2.845 | 0.092 |
|  | Yes | 436 (8.3) | 149 (2.8) |  |  |
| Animal care staff | No | 3630 (69.2) | 1452 (27.7) | 1.472 | 0.225 |
|  | Yes | 125 (2.4) | 40 (0.8) |  |  |
| Pet shop staff | No | 3742 (71.3) | 1484 (28.3) | 0.967 | 0.325 |
|  | Yes | 13 (0.2) | 8 (0.2) |  |  |
| Physiotherapist | No | 3575 (68.1) | 1432 (27.3) | 1.459 | 0.227 |
|  | Yes | 180 (3.4) | 60 (1.1) |  |  |
| Breeder | No | 3488 (66.5) | 1397 (26.6) | 0.918 | 0.338 |
|  | Yes | 267 (5.1) | 95 (1.8) |  |  |
| Podcasts | No | 3751 (71.5) | 1490 (28.4) | 0.071 | 0.790 |
|  | Yes | 4 (0.1) | 2 (0.0) |  |  |
| Magazines | No | 3750 (71.5) | 1491 (28.4) | 0.409 | 0.523 |
|  | Yes | 5 (0.1) | 1 (0.0) |  |  |
| Tv | No | 3742 (71.3) | 1487 (28.3) | 0.004 | 0.951 |
|  | Yes | 13 (0.2) | 5 (0.1) |  |  |
| Radio | No | 3754 | 1490 | 2.156 | 0.142 |
|  | Yes | 1 | 2 |  |  |
| **Friends family not vet** | No | **3222** | **1221** | **12.964** | **<0.001** |
|  | Yes | **533** | **271** |  |  |
| Friends family vet | No | 2942 | 1162 | 0.137 | 0.712 |
|  | Yes | 813 | 330 |  |  |
| Breed groups | No | 3157 | 1254 | 0.001 | 0.981 |
|  | Yes | 598 | 238 |  |  |
| Dog health group | No | 3215 | 1273 | 0.076 | 0.782 |
|  | Yes | 540 | 219 |  |  |
| **General dog group** | No | **3462** | **1344** | **6.214** | **0.013** |
|  | Yes | **293** | **148** |  |  |
| General groups | No | 3745 | 1490 | 0.819 | 0.366 |
|  | Yes | 10 | 2 |  |  |
| Scientific publications | No | 3744 | 1488 | 0.023 | 0.879 |
|  | Yes | 11 | 4 |  |  |
| Telemedicine | No | 3753 | 1491 | 0.035 | 0.851 |
|  | Yes | 2 | 1 |  |  |
| Webinars | No | 3753 | 1491 | 0.035 | 0.851 |
|  | Yes | 2 | 1 |  |  |

| 1^st^ Time Owner | No | 3311 (71.3) | 1333 (28.7) | 1.234 | 0.267 |
| --- | --- | --- | --- | --- | --- |
|  | Yes | 443 (73.5) | 160 (26.5) |  |  |
| Brachy Breed | No | 3130 (71.4) | 1253 (28.6) | 0.096 | 0.757 |
|  | Yes (Brachy or Brachy X) | 262 (72.2) | 101 (27.8) |  |  |
| Vet Registration | No | 25 (69.4) | 11 (30.6) | 0.078 | 0.780 |
|  | Yes | 3733 (71.6) | 1484 (28.4) |  |  |
| **Routine visits** | **Less than once a year** | **703 (64.6)** | **386 (35.4)** | **33.426** | **<0.001** |
|  | **Once a year or more** | **3025 (73.4)** | **1095 (26.6)** |  |  |
|  | **Miscellaneous** | **24 (68.6)** | **11 (31.4)** |  |  |
| **Vaccination** | **No** | **231 (66.8)** | **115 (33.2)** | **27.303** | **<0.001** |
|  | **Yes** | **2389 (74.1)** | **834 (25.9)** |  |  |
|  | **Yes – but not every year** | **1130 (67.6)** | **542 (32.4)** |  |  |
| **F+W Gold standard** | **No** | **1695 (66.9)** | **840 (33.1)** | **53.282** | **<0.001** |
|  | **Yes** | **2063 (76.0)** | **653 (24.0)** |  |  |
| **Long term condition** | **No** | **2662 (70.7)** | **1102 (29.3)** | **4.061** | **0.044** |
|  | **Yes** | **1088 (73.5)** | **392 (26.5)** |  |  |
| Owner Age | 18-24 years old | 113 (74.8) | 38 (25.2) | 9.466 | 0.221 |
|  | 25-34 years old | 432 (69.7) | 188 (30.3) |  |  |
|  | 35-44 years old | 544 (68.0) | 256 (32.0) |  |  |
|  | 45-54 years old | 873 (72.7) | 328 (27.3) |  |  |
|  | 55-64 years old | 1033 (72.0) | 402 (28.0) |  |  |
|  | 65-74 years old | 603 (72.7) | 226 (27.3) |  |  |
|  | 75-84 years old | 109 (73.6) | 39 (26.4) |  |  |
|  | Prefer not to say | 26 (78.8) | 7 (21.2) |  |  |
| Owner Gender | Female | 3514 (71.3) | 1412 (28.7) | 3.411 | 0.332 |
|  | Male | 177 (74.7) | 60 (25.3) |  |  |
|  | Other | 5 (55.6) | 4 (44.4) |  |  |
|  | Prefer not to say | 42 (77.8) | 12 (22.2) |  |  |
| Education | Level 1 / 2 | 668 (70.2) | 284 (29.8) | 8.113 | 0.422 |
|  | Level 3 | 724 (74.3) | 250 (25.7) |  |  |
|  | Level 4 | 12 (66.7) | 6 (33.3) |  |  |
|  | Level 5 | 22 (66.7) | 11 (33.3) |  |  |
|  | Level 6 | 1030 (70.2) | 438 (29.8) |  |  |
|  | Level 7 | 866 (71.9) | 338 (28.1) |  |  |
|  | Level 8 | 97 (70.3) | 41 (29.7) |  |  |
|  | Miscellaneous | 121 (75.6) | 39 (24.4) |  |  |
|  | Prefer not to say | 196 (71.0) | 80 (29.0) |  |  |
| Canine industry employment | No | 2984 (71.9) | 1164 (28.1) | 1.887 | 0.170 |
|  | Yes | 759 (69.8) | 328 (30.2) |  |  |
| Income | Up to £10,000 | 111 (76.0) | 35 (24.0) | 17.802 | 0.086 |
|  | £10,001 - £20,000 | 295 (70.2) | 125 (29.8) |  |  |
|  | £20,001 - £30,000 | 480 (71.0) | 196 (29.0) |  |  |
|  | £30,001 - £40,000 | 363 (68.6) | 166 (31.4) |  |  |
|  | £40,001 - £50,000 | 360 (72.4) | 137 (27.6) |  |  |
|  | £50,001 - £60,000 | 304 (67.4) | 147 (32.6) |  |  |
|  | £60,001 - £70,000 | 229 (70.9) | 94 (29.1) |  |  |
|  | £70,001 - £80,000 | 185 (79.4) | 48 (20.6) |  |  |
|  | £80,001 - £90,000 | 130 (71.4) | 52 (28.6) |  |  |
|  | £90,001 - £100,000 | 153 (73.9) | 54 (26.1) |  |  |
|  | More than £100,001 | 266 (74.9) | 89 (25.1) |  |  |
|  | Prefer not to say | 853 (71.4) | 342 (28.6) |  |  |
| **Insurance** | **No** | **1243 (67.9)** | **587 (32.1)** | **18.110** | **<0.001** |
|  | **Yes** | **2511 (73.5)** | **906 (26.5)** |  |  |
